# Grass and forbs respond differently to nitrogen addition: a meta-analysis of global grassland ecosystems

**DOI:** 10.1038/s41598-017-01728-x

**Published:** 2017-05-08

**Authors:** Chengming You, Fuzhong Wu, Youmin Gan, Wanqin Yang, Zhongmin Hu, Zhenfeng Xu, Bo Tan, Lin Liu, Xiangyin Ni

**Affiliations:** 1 Long-term Research Station of Alpine Forest Ecosystems, Provincial Key Laboratory of Ecological Forestry Engineering, Institute of Ecology and Forestry, Sichuan Agricultural University, 211 Huimin Road, Wenjiang District, Chengdu 611130 China; 2 Key Laboratory of Ecosystem Network Observation and Modeling, Institute of Geographical Sciences and Natural Resources Research, Chinese Academy of Sciences, Beijing, 100101 China; 3 College of Animal Science and Technology, Sichuan Agricultural University, 211 Huimin Road, Wenjiang District, Chengdu 611130 China

## Abstract

Nitrogen (N) deposition has increased globally and has profoundly influenced the structure and function of grasslands. Previous studies have discussed how N addition affects aboveground biomass (AGB), but the effects of N addition on the AGB of different functional groups in grasslands remain unclear. We conducted a meta-analysis to identify the responses of AGB and the AGB of grasses (AGB_grass_) and forbs (AGB_forb_) to N addition across global grasslands. Our results showed that N addition significantly increased AGB and AGB_grass_ by 31 and 79%, respectively, but had no significant effect on AGB_forb_. The effects of N addition on AGB and AGB_grass_ increased with increasing N addition rates, but which on AGB_forb_ decreased. Although study durations did not regulate the response ratio of N addition for AGB, which for AGB_grass_ increased and for AGB_forb_ decreased with increasing study durations. Furthermore, the N addition response ratios for AGB and AGB_grass_ increased more strongly when the mean annual precipitation (MAP) was 300–600 mm but decreased with an increase in the mean annual temperature (MAT). AGB_forb_ was only slightly affected by MAP and MAT. Our findings suggest that an acceleration of N deposition will increase grassland AGB by altering species composition.

## Introduction

Nitrogen (N) deposition in terrestrial ecosystems is estimated to increase to 200 Tg N yr^−1^ by 2050 due to industrial and agricultural N fertilizer use^1^. Nitrogen enrichment will potentially influence species diversity, biomass production and soil conditions^2–6^. The effects of N addition on forest ecosystem biomass have been summarized and analysed in previous studies^7–9^. However, because grasslands are mainly controlled by water, the effects of changes in precipitation patterns on aboveground biomass (AGB) were emphasized in previous studies^10–12^, and the effects of N addition on grassland biomass remain unknown. Grasslands are a type of terrestrial ecosystem and cover approximately 25% of the land surface on Earth^13^. AGB is an important contributor to soil organic matter, which significantly impacts the global carbon cycle under the background of N deposition^14, 15^. Therefore, analysing and summarizing the effects of N addition on grassland AGB are particularly important for estimating and predicting the carbon budget under climate change.

Many case studies that have been conducted to understand how N addition (N deposition) affects grassland AGB have yielded significantly different results^2, 16–18^. For example, several studies have reported significant increases^2, 18, 19^ and decreases^17^ or insignificant changes in AGB^16, 20^ following N addition. The differences between these results may be attributed to the use of different N addition rates, study durations, plant functional types and climatic conditions (such as the mean annual precipitation (MAP) or the mean annual temperature (MAT)). For instance, some previous studies have demonstrated a threshold value for the positive effects of N addition on AGB^2, 21^. If N application is greater than the threshold value, the positive effects will be reduced or disappear and can even cause metal toxicity, which will reduce AGB^22, 23^. Nevertheless, there is no global analysis of how experimental design parameters and climatic conditions regulate the response of AGB to N addition. Furthermore, adding N to grasslands can alter the soil nutrient^19, 24–26^ and water content^2^ and can reduce the soil pH^21^. Consequently, the effects of N addition on the soil environment and the subsequent AGB response remain controversial topics^2, 3, 19, 25^. Thus, analysing and discussing the mechanisms underlying the effect of N addition on AGB will provide a better understanding of how grassland AGB responds to N enrichment at the global scale.

Previous case studies found that different plant functional types in grasslands respond differently to N addition^3, 18, 26, 27^. For example, several studies have reported significant increases in the AGB of grasses and sedges^3, 23, 27^, significant decreases in the AGB of legumes^3, 27^, and insignificant changes in the AGB of forbs^27^ following N addition. However, whether a general pattern of the responses of different plant functional types in grasslands to N addition exists at the global scale remains unclear. Hence, further work is needed to determine the effects of N addition on the AGB of different functional groups in grasslands at the global scale.

The significantly different results from individual experiments are unlikely to reveal a general pattern that can be applied to global grasslands. In this study, we conducted a meta-analysis to quantitatively synthesize available studies on the changes in AGB, the AGB of grasses (AGB_grass_) and the AGB of forbs (AGB_forb_) in grasslands from around the world following N addition. Following previous comprehensive analyses of the effects of N addition on carbon and N cycles in terrestrial ecosystems^5, 28, 29^, we predicted that N addition would increase AGB, and N addition rates, study durations and climatic conditions would regulate the magnitude of the effect of N addition on AGB. To test these hypotheses, we asked the following questions: (i) What are the response patterns of AGB to N addition in grasslands around the world? Do all plant functional groups respond in a similar way? (ii) Do different N addition rates, study durations or climatic conditions influence the effects of N addition on AGB, AGB_grass_ and AGB_forb_?

## Results

### Effects of N addition on AGB and the soil environment

**Figure 1:**
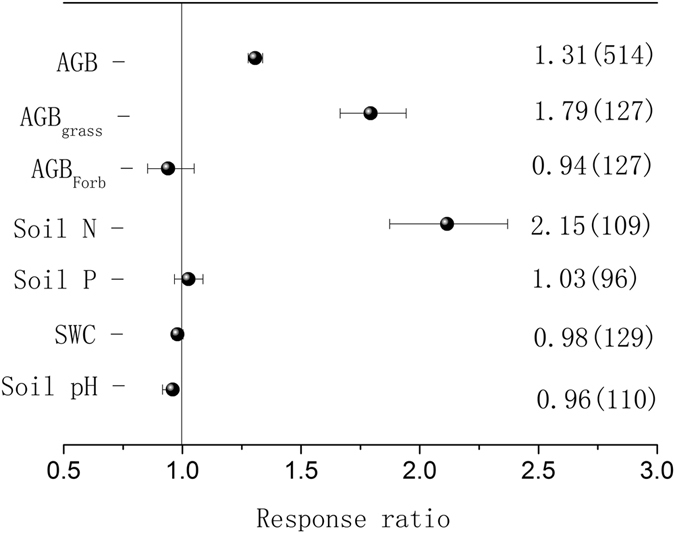
Effects of nitrogen (N) addition on aboveground biomass (AGB), the AGB of grasses (AGB_grass_), the AGB of forbs (AGB_forb_), soil available nitrogen (N) concentrations, soil available phosphorus (P) concentrations, soil water content, and soil pH. The numbers outside and inside the parentheses represent the response ratio (RR) and the number of observations, respectively. The dots with error bars are the means with 95% confidence intervals.

Across all of the studies at the global scale, N addition significantly increased AGB and AGB_grass_, with average increases of 31% and 79%, respectively, but did not significantly affect AGB_forb_ (Fig. 1). N addition significantly increased the soil available N concentrations by 115% but did not significantly affect the available P concentrations or the soil water content (Fig. 1). N addition also reduced the soil pH by an average of 4% (Fig. 1).

### Factors influencing the effects of N addition on AGB

**Figure 2:**
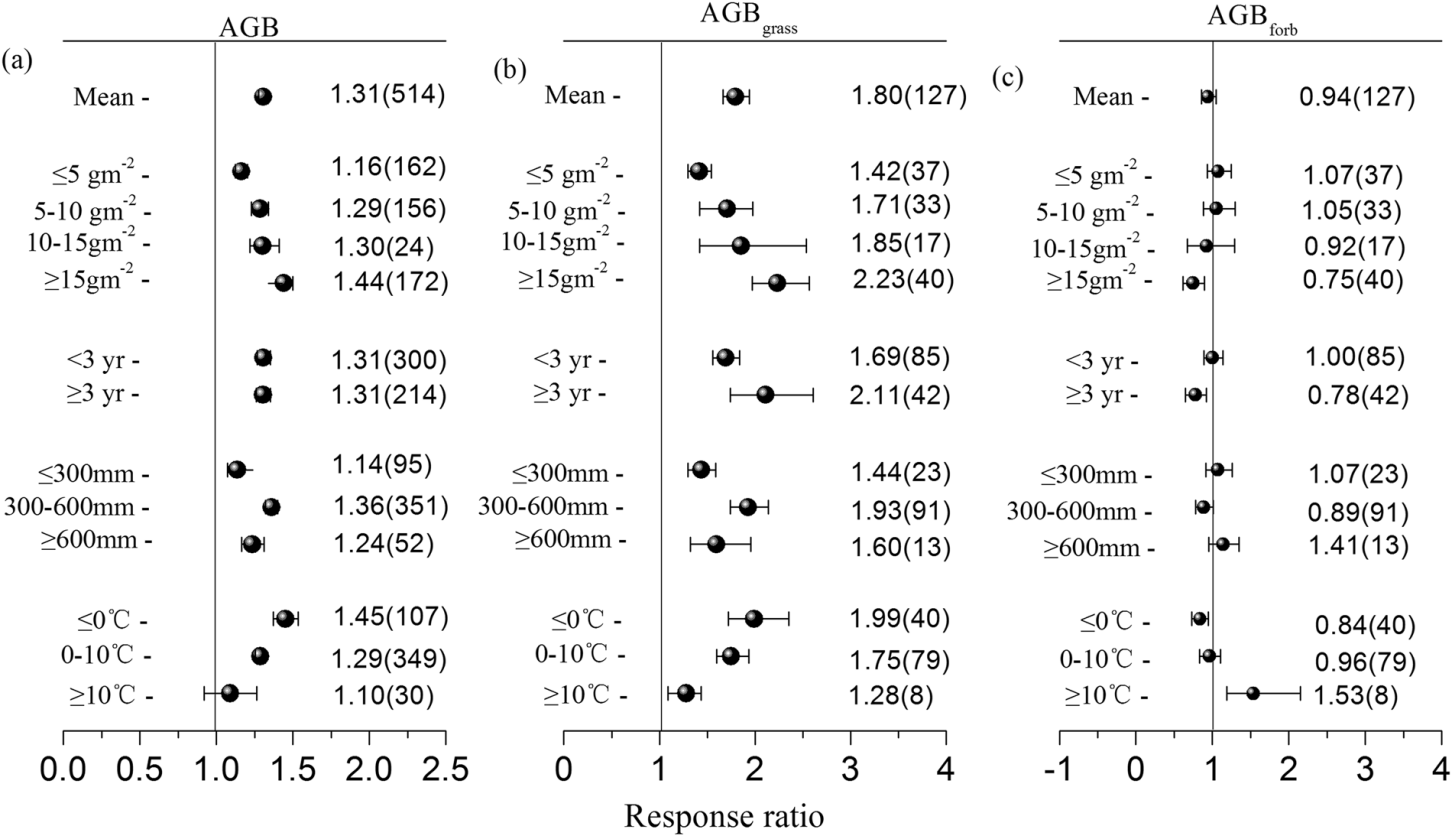
Effects of nitrogen (N) addition on aboveground biomass (AGB) (**a**), the AGB of grasses (AGB_grass_) (**b**), and the AGB of forbs (AGB_forb_) (**c**) under different N addition rates, study durations, and climatic conditions. The numbers outside and inside the parentheses represent the response ratio (RR) and the number of observations, respectively. The dots with error bars are the means with 95% confidence intervals.

Our results showed that the response ratios of AGB exhibited a quadratic function that changed with N addition rate (*p* < 0.001). From low to high N addition, the four N gradients significantly increased the AGB by an average of 16%, 29%, 30%, and 44% (all *p* < 0.05). The response ratios of AGB were not correlated with study duration (*p* = 0.899); however, the increase in AGB was significant for studies that were <3 years (31%) and those that were ≥3 years (31%), and no significant difference was observed between studies with durations <3 years and ≥3 years (*p* = 0.933). Moreover, the response ratios of AGB had weak correlations with MAP or MAT following N addition, but AGB changed significantly under their different groups (i.e., the increase was stronger when the MAP was 300–600 mm (36%) than when the MAP was ≥600 mm (24%) or ≤300 mm (14%) (*p* = 0.001) and decreased as the MAT increased (*p* = 0.001)) (Table 1, Fig. 2a).

**Table 1:** The relationships between the response ratios (RR) of the aboveground biomass (AGB), the AGB of grasses (AGB_grass_), the AGB of forbs (AGB_forb_) and the N addition rates, study durations, mean annual precipitation (MAP), and mean annual temperature (MAT) under nitrogen (N) addition based on a regression analysis of global grasslands.

	Equations	N	R^2^	*P*
RR of AGB				
N addition rates	y = −2.39 × 10^−4^x^2^ + 0.017x + 0.114	514	0.108	**0.001**
Study durations	y = −4.23 × 10^−4^x + 0.268	514	−0.002	0.899
MAP	y = −7.912 × 10^−7^x^2^ + 0.001x + 0.014	498	0.035	**0.001**
MAT	y = −0.007x + 0.289	485	0.006	**0.050**
RR of AGB_grass_				
N addition rates	y = 0.016x + 0.423	117	0.151	**0.001**
Study durations	y = 0.156x + 0.278	117	0.186	**0.001**
MAP	y = −8.889 × 10^−4^x + 1.013	117	0.030	**0.030**
MAT	y = −0.016x^2^ − 0.008x + 0.821	116	0.134	**0.001**
RR of AGB_forb_				
N addition rates	y = −0.016x + 0.059	117	0.085	**0.001**
Study durations	y = −0.202x + 0.307	117	0.165	**0.001**
MAP	y = 0.001x − 0.637	117	0.025	**0.049**
MAT	y = 0.015x^2^ + 0.037x − 0.368	116	0.079	**0.004**

Bold values indicate the significant portion of the variation among effect sizes that can be explained by an independent variable when *p* < 0.05.

The change trend of the response ratio of AGB_grass_ was similar to that of AGB under N addition. The response ratios of AGB_grass_ increased significantly as the N addition rates increased, and average increases of 42%, 71%, 85% and 123% were observed with increasing N gradient (all *p* < 0.05). When the data were subdivided by study duration, the response ratios of AGB_grass_ increased significantly as study durations increased (*p* < 0.001), and N addition had the most pronounced effect after 3 years (111%), which was more significant than the effects of N addition for less than 3 years (69%) (*p* = 0.015). The response ratios of AGB_grass_ had weak correlations with MAP or MAT under N addition. However, the effects of N addition on AGB_grass_ were larger when the MAP was 300–600 mm (93%) than when the MAP was ≤300 mm (44%) or ≥600 mm (60%) (*p* = 0.025) and decreased as the MAT increased (*p* = 0.068) (Table 1, Fig. 2b).

The change trend of the response ratios of AGB_forb_ was opposite to those of AGB and AGB_grass_ under N addition. The response ratios of AGB_forb_ decreased significantly as N addition increased, resulting in values of +7%, +5%, −8% and −25% was observed with increasing N gradient. In addition, the response ratios of AGB_forb_ decreased significantly as the study duration increased (*p* < 0.001); these values were significantly higher for study durations longer than 3 years (22%) than for study durations less than 3 years (*p* = 0.05). The response ratios of AGB_forb_ had weak correlations with MAP or MAT under N addition. N addition did not significantly affect AGB_forb_ for any MAP groups (all *p* > 0.05) in this study, but AGB_forb_ increased slightly as the MAT increased (*p* = 0.069) (Table 1, Fig. 2c).

### Factors influencing the effects of N addition on the soil environment

**Figure 3:**
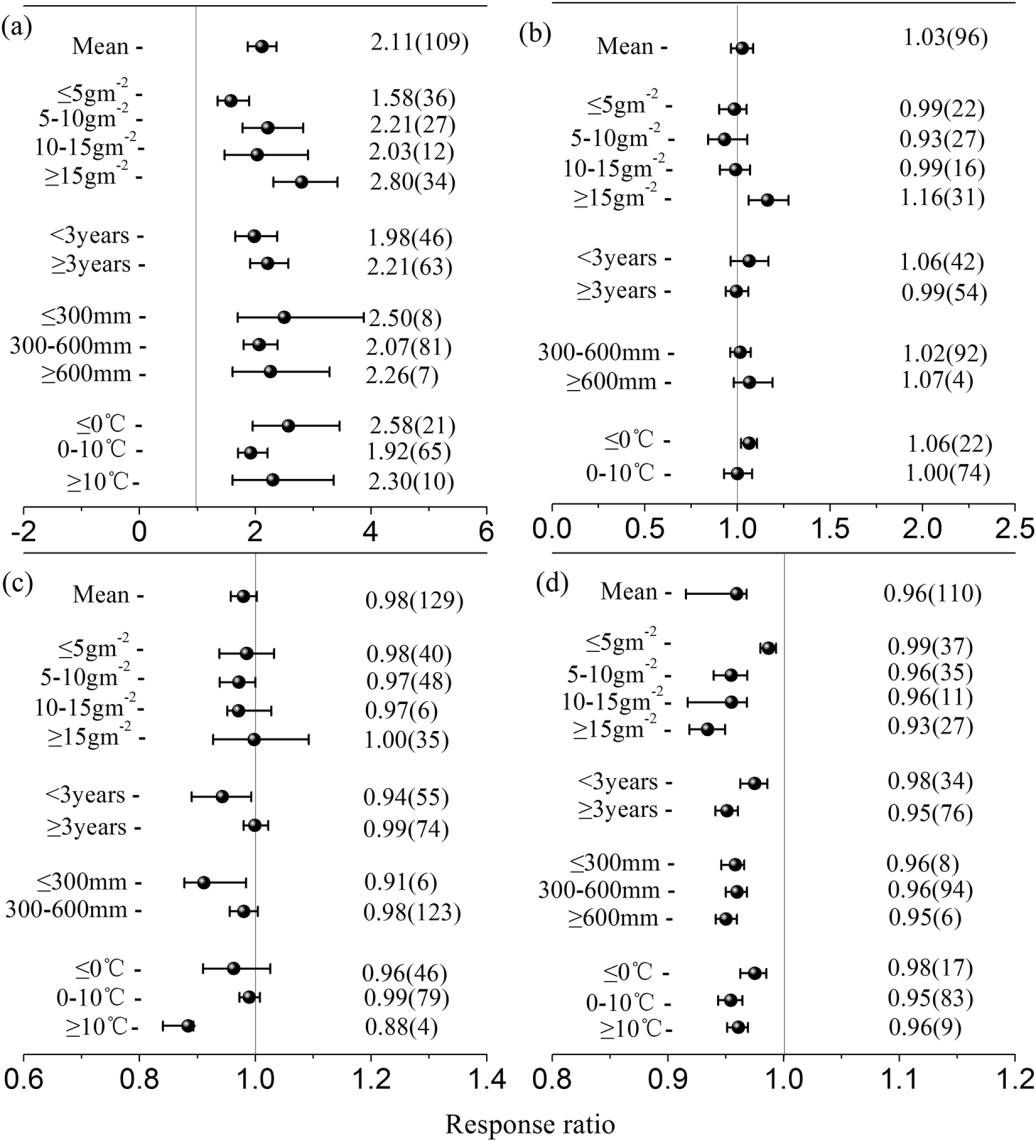
Effects of nitrogen (N) addition on soil available N concentrations (**a**), soil available phosphorus (P) concentrations (**b**), soil water content (**c**), and soil pH (**d**) under different N addition rates, study durations, and climatic conditions. The numbers outside and inside the parentheses represent the response ratio (RR) and the number of observations, respectively. The dots with error bars are the means with 95% confidence intervals.

In all of the considered studies, N addition increased the soil available N concentrations by an average of 115%. When the data were subdivided based on N addition rates, N addition increased the soil available N concentrations by 58%, 121%, 103%, and 180%, respectively, in the treatments with different N addition rates (in increasing order). When the data were subdivided by study duration and the MAP and MAT gradients, all of the indexes had significant effects on the soil available N concentrations, but no significant differences were observed among their subgroups (all *p* > 0.05) (Fig. 3a).

Overall, the soil available P concentrations did not indicate any significant changes due to N addition. Considering the investigated N addition rates, the most pronounced effect was observed at ≥15 g m^−2^ (16%), which was significantly greater than the effects observed in response to 10–15 g m^−2^ (−1%), 5–10 g m^−2^ (−7%), and ≤5 g m^−2^ (−1%) (*p* = 0.002). However, N addition had no significant effect on the soil available P concentrations across study duration and the MAP and MAT gradients (all *p* > 0.05) (Fig. 3b).

N addition had no significant effect on soil water content across all studies (Fig. 3c). The effect of N addition was significantly larger in studies with durations ≥3 years (−1%) than in studies with durations <3 years (−6%) (*p* = 0.03), but no significant differences were observed among the subgroups with different N addition rates or gradients of MAP or MAT (all *p* > 0.05) (Fig. 3c).

The soil pH significantly decreased by an average of 4% across all studies following N addition (Fig. 3d). The soil pH decreased with increasing N addition rate, with average decreases of 1%, 4%, 4% and 7% (all *p* < 0.05). The effect of N addition was pronounced for study durations ≥3 years (−5%) and was significantly greater for studies with durations ≥3 years than for studies with durations <3 years (−2%) (*p* = 0.005). N addition had a significantly negative effect on soil pH for all gradients of MAP and MAT, but no significant differences were observed among the MAP and MAT gradient subgroups (all *p* > 0.01) (Fig. 3d).

### The relationships between the response ratio of AGB and the soil environment

**Figure 4:**
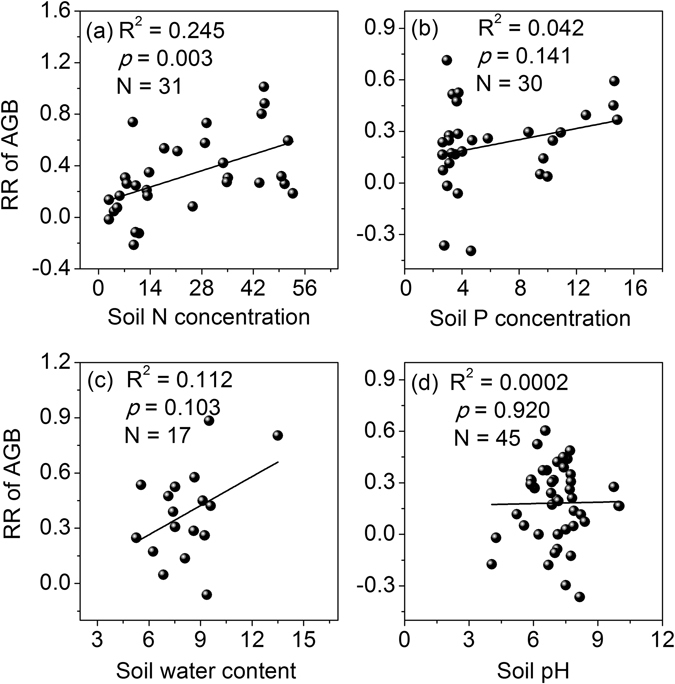
Relationships between the response ratio (RR) of the aboveground biomass (AGB) and the soil available nitrogen (N) concentrations (mg^−1^ kg) (**a**), soil available phosphorus (P) concentrations (mg^−1^ kg) (**b**), soil water content (v/V%) (**c**), and soil pH (**d**).

The response ratio of AGB changed with soil environmental factors. As the soil available N concentrations increased, the response ratio of AGB increased significantly (*p* = 0.003, Fig. 4a). The response ratio of AGB increased linearly with soil available P concentrations (Fig. 4b) and soil water content (Fig. 4c); however, neither of these relationships were significant (all *p* > 0.01). In addition, the response ratio of AGB was not significantly correlated with changes in soil pH (Fig. 4d).

## Discussion

### Effects of N addition on AGB, AGB_grass_ and AGB_forb_

Nitrogen has been reported to limit biomass production in most previous summaries of grassland ecosystem research^9, 30–33^. Our results demonstrated consistent and statistically significant responses of grassland AGB to N addition (Figs 1 and 2), which is similar to the results presented in previous studies^4, 9, 30, 31, 34^. Notably, the response ratio of AGB observed in our study (31%) is comparable to the synthesis of grassland studies (31.7%^14^ and 30%^34^) but is lower than the response ratios observed in other studies^4, 9, 30, 31^. Two reasons may explain this phenomenon. First, the different response ratios may be attributed to different ecosystem types: grasslands are the only ecosystem type considered in our meta-analysis, whereas previous studies include most types of terrestrial ecosystems^4, 30^. In addition, LeBauer & Treseder^9^ showed that the response ratio of tropical forests was larger than that of tropical grasslands. Therefore, these findings indicate that the response ratio is larger in forests than in grasslands under N deposition. Second, the different response ratios can be attributed to different management types; for example, Yahdjian *et al*.^31^ showed that the response ratio of natural grasslands was lower than that of sown pastures. In addition, our study only includes natural ecosystems, and previous studies include artificial ecosystems^31^.

N addition can promote photosynthesis by boosting foliar N concentrations, and significantly increasing AGB^35^, which can be attributed to changes in the soil physical or chemical properties, such as enhanced soil available N^3, 25, 26^, soil available P^19, 36^ or soil moisture^2^. In our study, the response ratio of AGB was positively correlated with the soil available N (Fig. 4a), but it was not significantly correlated with the changes in soil available P (Fig. 4b) or soil water content (Fig. 4c). These results indicate that the enhanced AGB is mainly attributable to increased soil available N under N addition, which is supported by previous studies^3, 26, 37^.

Our results also showed that AGB_grass_ increased with N addition, but AGB_forb_ remained unchanged. These results indicate that the increase in AGB occurred through increasing AGB_grass_ and not AGB_forb_, which is similar to previous studies^3, 18, 27^. Three mechanisms may support this phenomenon. The first is competition for light and space. Most grasses are taller than forbs and can occupy a larger space to capture more light for growth when N is added^24, 38^. A previous study also reported that competition for light led to a loss of plant biodiversity resulting from eutrophication^39^. The second mechanism relates to competition for soil resources. Most grasses have highly branched fibrous root systems that are mainly distributed near the soil surface and may have the advantage of absorbing more nutrients from the soil surface and using these nutrients more effectively (including soil N^40^, P^41^, and Ca^2+24^). Third, soil acidification results in metal toxicity in forbs. A recent study showed that N addition led to soil acidification and released more manganese ions (Mn^2+^), resulting in greater accumulation of Mn^2+^ in forbs than in grasses and ultimately a significant reduction in forbs photosynthesis and growth^23^. Although we did not directly collect metal ion data, our results showed that N addition significantly reduced the soil pH. A global study demonstrated that decreased soil pH could result in the release of large amounts of Mn^2+^ and aluminium (Al^3+^) ions and limited plant growth^22^. This general pattern indicates that plant functional types have different responses to N addition and suggests that significant acceleration of atmospheric N deposition will remarkably impacts the structure and stability of grassland ecosystems.

### Factors influencing the effects of N addition

This synthesis of all considered experiments showed that the response ratio of AGB to N addition was influenced by the N addition rate (Fig. 2). Specifically, this response ratio increased as the N application level increased, which is consistent with previous case studies^2, 18, 24^ and synthesis studies^3, 42^. This finding potentially occurred because high levels of N addition can provide more soil available N^5,37^ (Fig. 3a) and can improve the status of other soil nutrients^36^ (Fig. 3b). Furthermore, our results showed that the effects of N addition on AGB_grass_ increased as the rate of N increased, while AGB_forb_ decreased as the rate of N increased, which is supported by some previous studies^23, 26, 27^. These results may be due to the compensatory effect between AGB_grass_ and AGB_forb_ under N addition^18, 24, 43^. Increases in N addition gradients can lead to decreases in soil pH and the release of heavy metal elements, which can significantly reduce forbs diversity^23^. In addition, increasing N rates can supply more soil available N, P and water, which are used more effectively by grasses^41, 43^. Our results also showed that AGB_grass_ and soil available N or P undergo similar increasing trends with increasing N addition rates (Fig. 3). These findings indicate that a threshold value exists for the positive effects of N addition on AGB; if N addition exceeds the threshold value, the positive effects of increasing the rate of N will decrease or disappear.

Study duration did not regulate the response ratio of AGB to N addition, and the AGB_grass_ and AGB_forb_ N addition response ratios increased and decreased, respectively, with increasing study duration. These results can be explained by the following reasons. First, N addition ensures that the ecosystem is not limited by N and promotes plant growth^2^. However, with increasing study duration, the grasses will limit the growth of the forbs because grasses in the upper layer of an ecological niche can better capture other resources (light^24^, etc.), and the forbs have slower growth rates^44^. Second, soil acidification would become more serious with an increase in study duration^22, 23^ (Fig. 3d), which may aggravate metal toxicity and curtail forbs growth^23^. Furthermore, previous studies demonstrated that forbs were gradually replaced by grasses with increasing study duration^18, 24^, which is supported by our results.

In addition to study duration and N addition rate, climate factors also influenced the effects of N addition on AGB, AGB_grass_ and AGB_forb_. The response ratio of AGB to N addition increased more strongly when the MAP was 300–600 mm. This is similar to a previous meta-analysis^30^, which reported that the greatest effect of N addition on AGB occurred under approximately 500 mm of rainfall and when the MAP was <1200 mm. These findings suggest that the growth of grassland plants is co-limited by N and water availability (Fig. 2a). In our study, water deficiency may have inhibited the uptake of N by plants and suppressed the positive effects of N addition on plant growth^43, 45^. As annual precipitation increases, soil moisture increases and results in the soil releasing more N, ultimately promoting plant growth^46^. However, when annual precipitation exceeds approximately 600 mm, water limitations may disappear but the positive effects of N addition on plant growth may be influenced by other confounding factors, such as N loss^6^ and temperature^2^. The response ratio of AGB_grass_ to N addition increased more strongly when the MAP was 300–600 mm, but the MAP only slightly affected the response ratio of N addition for AGB_forb_. As discussed above, the response of AGB_grass_ to N addition is more sensitive than AGB_forb_^2, 30^ since the changes in AGB_grass_ are mainly determined by soil N. However, the changes in AGB_grass_ will correspond regulate the response of AGB_forb_ to N addition^18, 24, 43^.

The magnitude of the effect of N addition on AGB was larger when MAT was ≤0 °C compared with 0–10 °C and ≥10 °C (Fig. 2a). This is similar to a previous meta-analysis^30^, which reported that the greatest effects of N addition on AGB occurred at approximately ≤0 °C and when the MAT was <15 °C. However, the previous meta-analysis also found that the effects of N addition on AGB decreased when the MAT increased and when the MAT was >15 °C (the effects were greater when the MAT was >15 °C than that when the MAT was <15 °C)^30^. Notably, a small number of sites had MAT >15 °C in our study; we did not divide this group because it would have limited the statistical power of the analysis. These above-mentioned results may be explained by warming, which can increase the temperature^47^ and the evapotranspiration of ecosystems^48^, leading to water deficiency and accelerating acid production in soil^49^ under N addition. Finally, it restrains plant growth and decreases the response of AGB to N addition. Furthermore, the response ratio of AGB_grass_ to N addition decreased with increasing MAT compared with an increase for AGB_forb_. This result may have occurred because warming increases the competitive ability of forbs while suppressing the competitive ability of grasses^50^.

### Suggestions for simulating N deposition in the future

Although multiple N deposition simulation experiments have been conducted, these studies may not completely imitate actual N deposition because the studied N addition rates, study durations, N-addition treatments, fertilization times and climatic conditions were different from the actual conditions. According to this meta-analysis, we provide some constructive suggestions for experiments on the effects of N added to grasslands considering the following four aspects. First, most of the experimental levels of N were much higher than the natural N deposition rates (approximately <5 g m^−2^ yr^−1^)^21^, which would overestimate the effects of N deposition on AGB, AGB_grass_ and AGB_forb_ and caused some serious soil environmental problems in grasslands^22^. Second, N deposition is usually accompanied by phosphate deposition or precipitation and is regulated by other factors^51^ (such as elevated Carbon dioxide and warming). However, fewer studies have focused on the effects of N addition interaction with other factors (such as P and water) on AGB, AGB_grass_ and AGB_forb_, when comparing with the treatment of N applied alone (Fig. S3). These results indicate that experimental designs consisting of multiple factors are somewhat lacking, and further studies should focus on the effects of N addition on AGB considering multiple factors to create a model that provides better predictions and that can be used for grassland conservation. Third, fertilizer was added less than 3 times during the growing season in most of the experiments with N addition and did not significantly change the effects of N addition on AGB (Fig. S3). This is consistent with a previous case study^52^. However, some studies found that using a wide range of number of N fertilization applications had a significant impact on grassland ecosystem structure and function^53, 54^. These findings suggest that additional N applications and N application during the non-growing season may have large effects on AGB in long-term experiments. Fourth, although we have done our best to collect data on the impacts of N addition on AGB around the world, most of our data were obtained from international journals and consisted of studies conducted on Tibetan Plateau or Inner Mongolian grasslands published by Chinese or international scholars (Table S1). Similar with other meta-analysis, the distribution of sample sites is always the flaw which limits our results. Therefore, new experiments with appropriate N addition rates, combined with other factors and a wide range of fertilization times and sites, should be considered to better understand how N deposition influences grasslands in the future.

## Conclusions

We conducted a meta-analysis to identify the general patterns of the effects of N addition on AGB, AGB_grass_, and AGB_forb_ and considered that the variations of these effects relative to the major factors controlling the strength of the response to N addition are important for understanding the effects of altered N inputs on global grasslands. First, our results showed that N addition significantly increased AGB and AGB_grass_ but had no significant effect on AGB_forb_. These suggest that N deposition will likely enhance the dominant position of grasses in grasslands and will change their structure and stability under global climate change. Second, the N addition response ratios of AGB, AGB_grass_, and AGB_forb_ also varied with N addition rate, study duration and climatic conditions. Thus, we conclude that the results from short-term experiments and single doses or N addition alone may not accurately predict the long-term effects of N deposition on grassland productivity. Our results also revealed that the N addition response ratio of AGB increased as the soil available N concentrations increased, indicating that soil available N is an important factor that determines how N addition affects grassland AGB. The findings of this study suggest that significant increases in atmospheric N deposition will increase grassland AGB by altering its structure and the soil environment under global climate change.

## Materials and Methods

### Data selection

Peer-reviewed journal articles were searched using Web of Science and China National Knowledge to compile a database, which included the responses of AGB, AGB_grass_, AGB_forb_, soil available N and P, soil water content, and soil pH to N addition. The following key words were used to identify studies: N addition/deposition/enrichment, N + P addition, N + Water (W) addition, biomass production, AGB, aboveground net primary production, soil available N and P, and grasslands. To minimize publication bias, only previous case studies that satisfied the following criteria were selected for inclusion in the database for analyses. (i) The experimental data must be collected from field experiments involving N addition and conducted in natural grasslands. (ii) Experiments must include N-addition treatments and a control treatment; if the field experiment only had N addition as a treatment, the original experimental control was used to estimate the response ratio of only N addition for AGB, AGB_grass_ and AGB_forb_; if the N-addition treatments were combined with P or W, the treatments with only P or W addition were regarded as the control treatments to eliminate the effects of other factors on AGB, AGB_grass_ and AGB_forb_. (iii) AGB was measured during the peak growing season, and these data were compiled into a database; data not obtained during the peak growing season were excluded. (iv) If experimental data from a particular site was published in different journal articles, the most recent paper or the paper with the longest record was chosen. (v) The means, sample sizes, standard deviations (SDs) and/or standard errors (SEs) of the treatments and controls were collected or calculated for each case study. The data were obtained from 89 peer-reviewed journal articles and corresponded to 67 sites. Among these studies, 18 included N + P addition, 15 included N + W addition, and 56 included only N addition.

The raw data were directly obtained from tables in the journal articles or were extracted from graphs using the Get Data Graph Digitizer (version 2.24, Russian Federation) (The response ratios of N addition for AGB, AGB_grass_, and AGB_forb_, are provided as supplementary information). For detailed analyses and comparisons, we also collected background information relevant to the data from the papers. For instance, the N addition rates, study durations, experimental years, fertilization times, fertilization types and the latitude, longitude, mean annual precipitation (MAP), and mean annual temperature (MAT) of the sites were obtained from the journal articles. If the published papers did not directly show the MAP or MAT, these data were extracted from the *WorldClim* database (http://www.worldclim.org) based on the name or longitude and latitude of the study location. To determine how the experimental designs and climatic conditions impacted the response ratios of AGB, AGB_grass_, and AGB_forb_ to N addition, we grouped the data into N addition rate (≤5, 5–10, 10–15, and ≥15 g m^−2^), study duration (<3 years and ≥3 years), N-addition treatment (only N, N + P, and N + W addition), number of fertilizer applications (1, 2, and 3 times), MAP (≤300, 300–600, and ≥600 mm), and MAT (≤0, 0–10, and ≥10 °C) based on previous studies^2, 4, 21, 30, 35^. According to Tian *et al*.^22^, the effects of NH_4_NO_3_ and NH_4_ fertilizer on AGB were not significantly different from each other and consequently, were not discussed.

### Meta-analysis

The data were analysed using the meta-analysis approach described by Hedges *et al*.^55^. The N addition amount for each individual observation was estimated using the unlogged effect size results and the response ratio (RR) as follows: ln RR = In (*X*_*T*_/*X*_*C*_), where *X*_*T*_ is the treatment mean and *X*_*C*_ is the control mean. Mixed modelling was conducted using the meta-analytical software Meta-win 2.1 (Sinauer Associates, Inc. Sunderland, MA, USA) to calculate the weighted response ratio and the 95% confidence interval (95% CI). The 95% CI was also used to determine whether the weighted N addition response ratio was significant for a specific variable. If the bounds of the 95% CI of the response ratio overlapped by 1, the response ratio of the variable was not significant for that treatment. If the end of the 95% CI was greater or less than 1, the response ratio of the variable for that treatment was considered significantly positive or negative at *p* < 0.05. The detailed components (such as the variance, weighted response ratio, and 95% confidence interval) used in this calculation method are described in detail in peer-reviewed journal articles^3, 4^.

To determine whether the experimental design or climatic conditions influenced the effects of N addition, the data were subdivided as described above. To test for significance between the subgroups, the method described by Hedges *et al*.^55^ was used to calculate the total heterogeneity, the within-group heterogeneity and the between-group heterogeneity. The total heterogeneity was also partitioned into within- and between-group heterogeneity, and significance of the between-group heterogeneity indicated that the response ratios differed among different subgroups. If the bounds of the 95% CI of the means of the subgroups did not overlap by 1, they were considered significantly different from each other.

### Sensitivity analysis and publication bias

We conducted a sensitivity analysis to determine how robust the results were based on the decisions and assumptions made in the meta-analysis (the data structure can take a variety of forms)^56^, and a mixed model was used to calculate the weighted response ratio. In further tests, we randomly sampled 5 data sets (samples sizes of 100, 200, 300, 400, and 500) from the total AGB sample (Table S1) and then used the mixed model to reanalyse the weighted response ratio (sensitivity analyses of AGB_grass_ and AGB_forb_ were also performed using this method, Fig. S1). The results obtained from the 5 groups were the same as those obtained from the total sample (Fig. S1). This finding suggests that reducing the number of samples will not change the results of this study. Publication bias testing is also a form of sensitivity analysis^56^. We tested for publication bias, which is the selection of a section of published articles that leads to trial bias, by observing whether the data followed a normal distribution^57^. This test indicated no publication bias (AGB, AGB_grass_, and AGB_forb_ were all normally distributed Fig. S2). Some previous studies also indicated that using a large amount of data reduces the occurrence of publication bias^9^.

### Statistical analysis

The relationships between the response ratio of AGB and the experimental design parameters and soil and climatic conditions were examined. Specifically, regression analysis was used to analyse the relationships between the response ratio of AGB and the N addition rates, study durations, climatic factors, soil available N, soil available P, soil water content and soil pH. SPSS software (SPSS 17.0 for windows; SPSS Inc., Chicago, IL, USA) was used for regression and correlation analyses, and graphs were drawn using Origin (version 8.0).

## Electronic supplementary material

SREP-16-39526A_Supplementary Information
