# Supplementary material for: Grass and forbs respond differently to nitrogen addition: a meta-analysis of global grassland ecosystems
Source: Sci Rep. 2017 May 8;7:1563. doi: 10.1038/s41598-017-01728-x (PMC5431500; doi:10.1038/s41598-017-01728-x)
Supplement: Supplementary file 1 — SREP-16-39526A_Supplementary Information [file 41598_2017_1728_MOESM1_ESM.doc]

**SUPPLEMENTARY INFORMATION TO**

**Grass and forbs respond differently to nitrogen addition: a meta-analysis of global grassland ecosystems**

Chengming You^1,2,3^, Fuzhong Wu^1^, Youmin Gan^3^, Wanqin Yang^1^*, Zhongmin Hu^2^*, Zhenfeng Xu^1^, Bo Tan^1^, Lin Liu^3^& Xiangyin Ni^1^

Affiliation:

^1^Long-term Research Station of Alpine Forest Ecosystems, Key Laboratory of Ecological Forestry Engineering, Institute of Ecology and Forestry, Sichuan Agricultural University, Chengdu 611130, China. ^2^Key Laboratory of Ecosystem Network Observation and Modeling, Institute of Geographical Sciences and Natural Resources Research, Chinese Academy of Sciences, Beijing 100101, China. ^3^College of Animal Science and Technology, Sichuan Agricultural University, Chengdu 611130, China.

*Corresponding author

**Supplementary Information**

**Fig. S1 Repeat analysis of the effects of nitrogen (N) addition on aboveground biomass (AGB) (a), the AGB of grass (AGB_grass_) (b), and the AGB of forbs (AGB_forb_) (c) under different sample sizes.**

**Fig. S2 Frequency distribution of the RR of nitrogen (N) addition on aboveground biomass (AGB) (a), the AGB of grass (AGB_grass_) (b), and the AGB of forbs (AGB_forb_) (c).** The curves were fitted by a Gaussian function.

**Fig. S3 Effects of nitrogen (N) addition on aboveground biomass (AGB) (a), the AGB of grass (AGB_grass_) (b), and the AGB of forbs (AGB_forb_) (c) in the different N-addition treatments and for different fertilization times.** The numbers outside and inside parentheses represent the RR and the number of observations. The dots with error bars show the means with the 95% confidence intervals.

**Fig. S4 Effects of nitrogen (N) addition on soil available N concentrations (a), soil available phosphorus (P) concentrations (b), soil water content (c), and soil pH (d) in the different N-addition treatments and for different fertilization times.** The numbers outside and inside the parentheses represent the RR and the number of observations. The dots with error bars show the means with the 95% confidence intervals.

Figure. S1


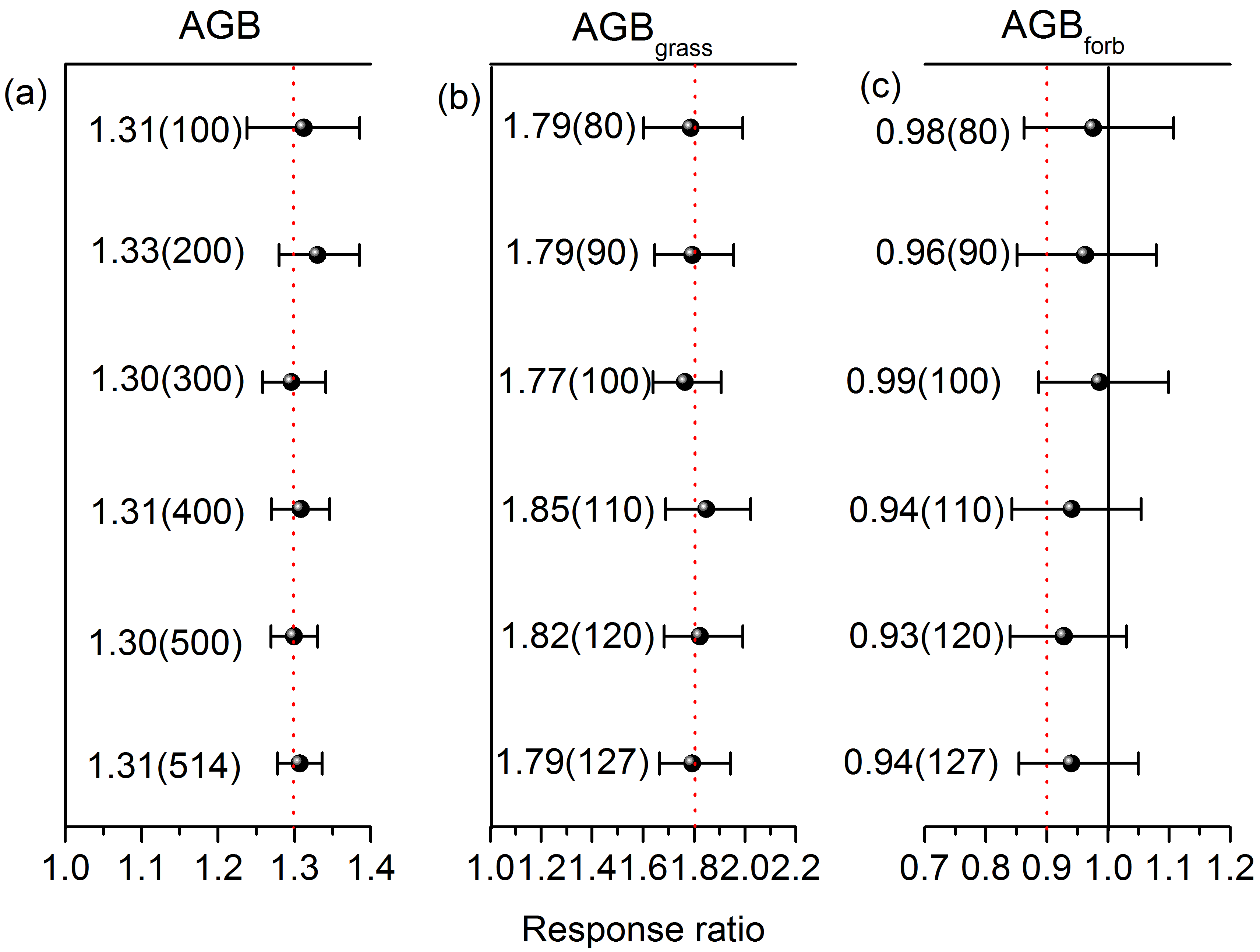


Figure. S2


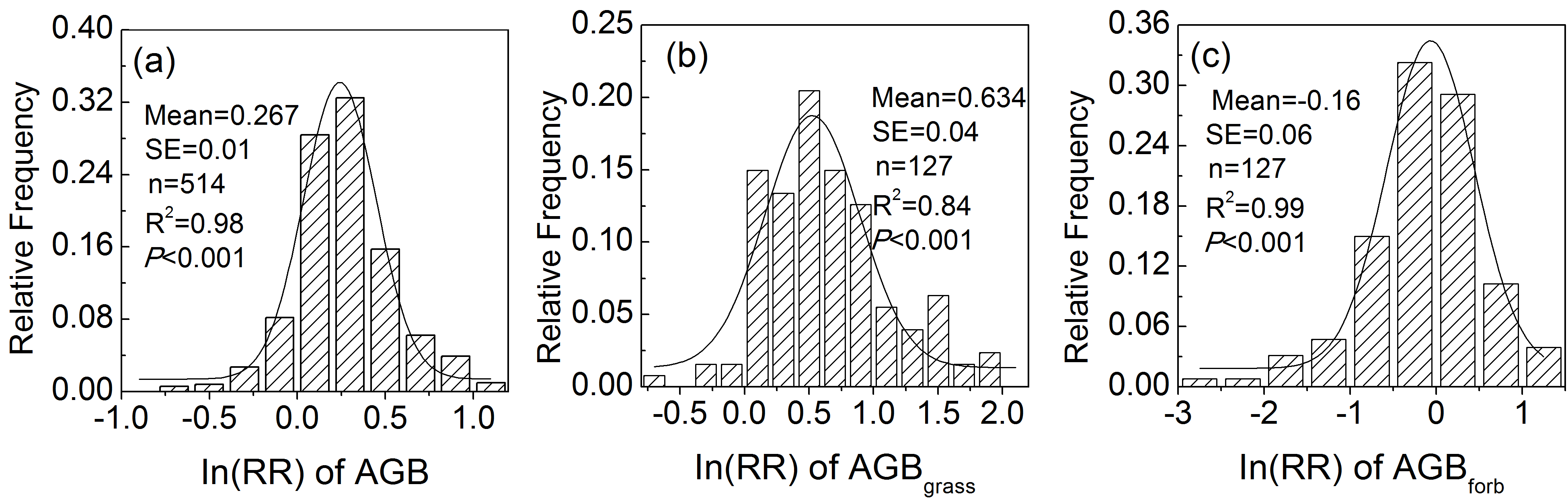


Figure. S3


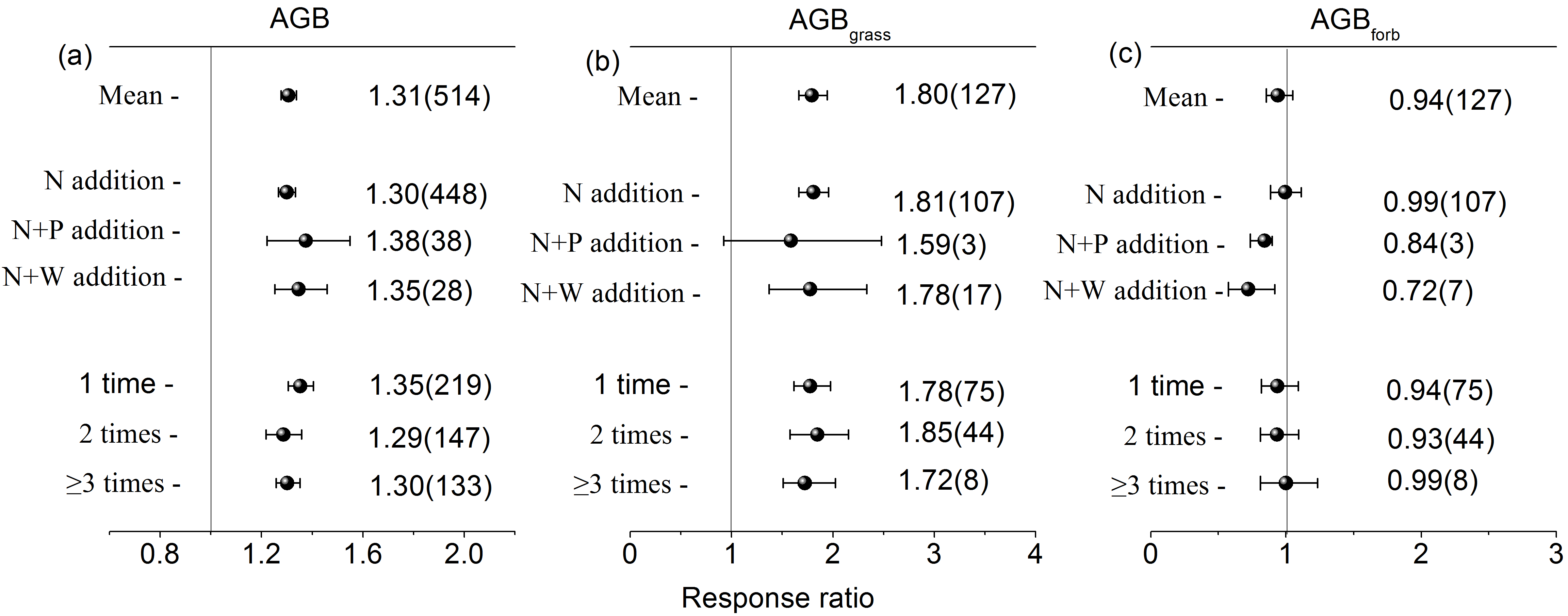


Figure. S4


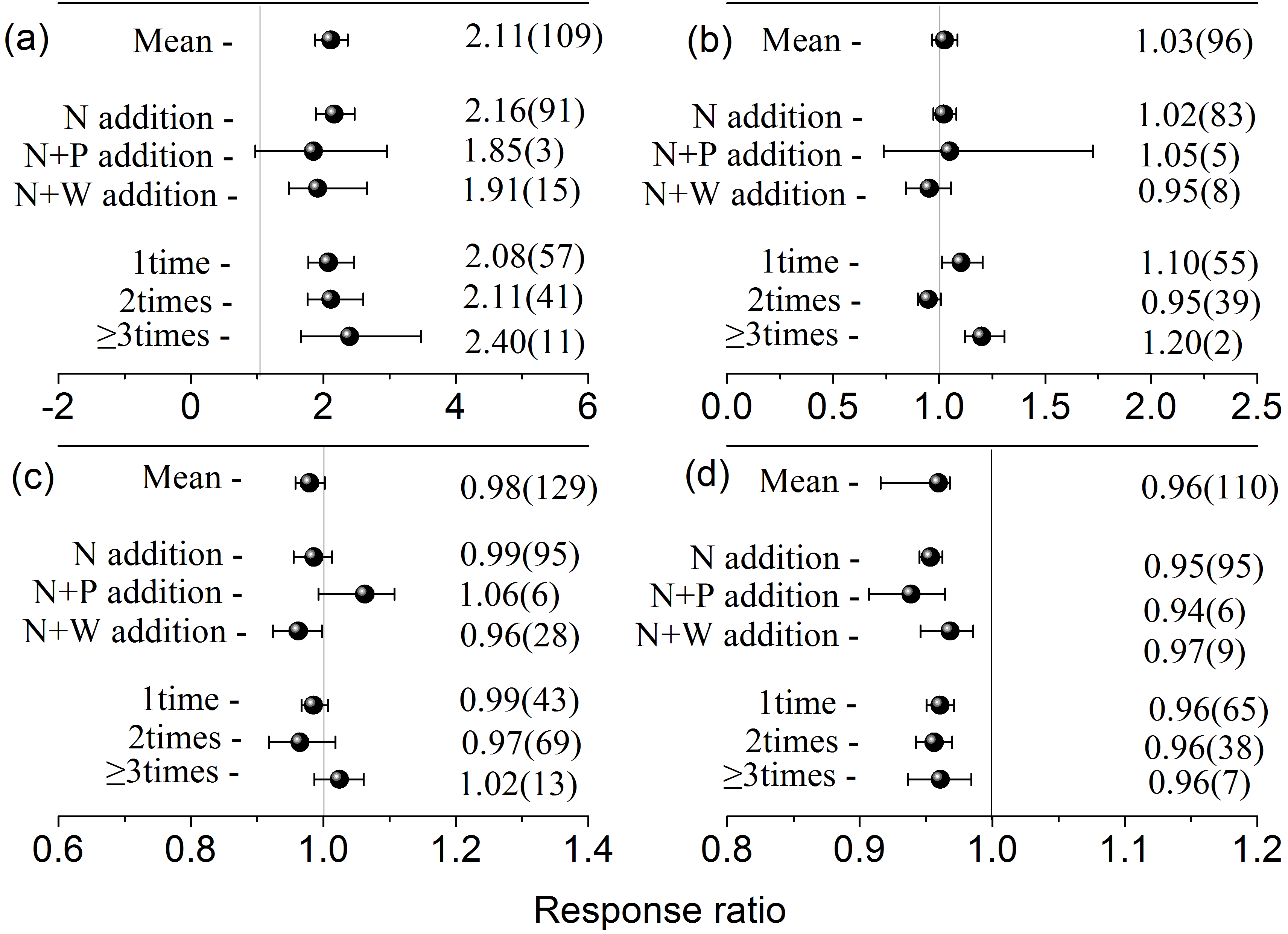


**Table S1 The papers included in this study for establishing the database of the effects of N addition on aboveground biomass (AGB), the AGB of grass (AGB_grass_), and the AGB of forbs (AGB_forb_).**

| n | Author | Grassland types | longitude | latitude | year | treatment | AGB | AGB_grass_ | AGB_forb_ |
| --- | --- | --- | --- | --- | --- | --- | --- | --- | --- |
| 1 | Tianhong Zhu | alpine meadow | 101°19'E | 37°37'N | 2009 | N1 | 0.209 |  |  |
| 2 | Tianhong Zhu | alpine meadow | 101°19'E | 37°37'N | 2009 | N2 | 0.056 |  |  |
| 3 | Tianhong Zhu | alpine meadow | 101°19'E | 37°37'N | 2009 | N4 | 0.056 |  |  |
| 4 | Tianhong Zhu | alpine meadow | 101°19'E | 37°37'N | 2009 | N1 | 0.239 |  |  |
| 5 | Tianhong Zhu | alpine meadow | 101°19'E | 37°37'N | 2009 | N2 | 0.103 |  |  |
| 6 | Tianhong Zhu | alpine meadow | 101°19'E | 37°37'N | 2009 | N4 | 0.175 |  |  |
| 7 | Tianhong Zhu | alpine meadow | 101°19'E | 37°37'N | 2009 | N1 | 0.197 |  |  |
| 8 | Tianhong Zhu | alpine meadow | 101°19'E | 37°37'N | 2009 | N2 | 0.245 |  |  |
| 9 | Tianhong Zhu | alpine meadow | 101°19'E | 37°37'N | 2009 | N4 | 0.223 |  |  |
| 10 | Zhen Wang | desert steppe | 111°53′E | 41°47′N | 2006 | N10 | -0.322 |  |  |
| 11 | Zhen Wang | desert steppe | 111°53′E | 41°47′N | 2007 | N10 | 0.187 |  |  |
| 12 | Zhen Wang | desert steppe | 111°53′E | 41°47′N | 2008 | N10 | 0.134 |  |  |
| 13 | Zhen Wang | desert steppe | 111°53′E | 41°47′N | 2009 | N10 | 0.101 |  |  |
| 14 | Ning Zong | alpine meadow | 91°05′E | 30°51′N | 2011 | N4 | 0.19 |  |  |
| 15 | Ning Zong | alpine meadow | 91°05′E | 30°51′N | 2012 | N4 | 0.235 |  |  |
| 16 | Xiaodong Liu | alpine meadow | 102°24.1' E | 35°05.6' N | 2014 | N5 | 0.028 |  |  |
| 17 | Xiaodong Liu | alpine meadow | 102°24.1' E | 35°05.6' N | 2014 | N10 | 0.146 |  |  |
| 18 | Xiaodong Liu | alpine meadow | 102°24.1' E | 35°05.6' N | 2014 | N15 | -0.059 |  |  |
| 19 | Yuejuan Yang | alpine meadow | 101°12' E | 37°29' N | 2010 | N36 | 0.182 |  |  |
| 20 | Yuejuan Yang | alpine meadow | 101°12' E | 37°29' N | 2010 | N36P | 0.26 |  |  |
| 21 | Li Yu | Meadow steppe | 119°42' E | 48°30' N | 2012 | N10 | 0.448 |  |  |
| 22 | Li Yu | Meadow steppe | 119°42' E | 48°30' N | 2012 | N10P | 0.268 |  |  |
| 23 | Rui Qi | Sub alpine meadow | 102°00' E | 35°00' N | 2012 | N5 | 0.23 |  |  |
| 24 | Rui Qi | Sub alpine meadow | 102°00' E | 35°00' N | 2012 | N10 | 0.27 |  |  |
| 25 | Rui Qi | Sub alpine meadow | 102°00' E | 35°00' N | 2012 | N15 | 0.411 |  |  |
| 26 | Rui Qi | Sub alpine meadow | 102°00' E | 35°00' N | 2012 | N5P | 0.436 |  |  |
| 27 | Rui Qi | Sub alpine meadow | 102°00' E | 35°00' N | 2012 | N10P | 0.859 |  |  |
| 28 | Rui Qi | Sub alpine meadow | 102°00' E | 35°00' N | 2012 | N15P | 0.992 |  |  |
| 29 | Yuhui He | Typical desert steppe | 103°47' E | 36°13' N | 2013 | N1 | 0.511 |  |  |
| 30 | Yuhui He | Typical desert steppe | 103°47' E | 36°13' N | 2013 | N5 | 0.385 |  |  |
| 31 | Yuhui He | Typical desert steppe | 103°47' E | 36°13' N | 2013 | N10 | 0.406 |  |  |
| 32 | Fei Han | Sub alpine meadow | 102°53' E | 34°55' N | 2013 | N5 | 0.251 |  |  |
| 33 | Fei Han | Sub alpine meadow | 102°53' E | 34°55' N | 2013 | N10 | 0.465 |  |  |
| 34 | Fei Han | Sub alpine meadow | 102°53' E | 34°55' N | 2013 | N15 | 0.469 |  |  |
| 35 | Jieqiong Su | desert steppe | 105°1'47.99"E | 37°31'48"N | 2011 | N1.75 | -0.305 |  |  |
| 36 | Jieqiong Su | desert steppe | 105°1'47.99"E | 37°31'48"N | 2011 | N3.5 | -0.776 |  |  |
| 37 | Jieqiong Su | desert steppe | 105°1'47.99"E | 37°31'48"N | 2011 | N7 | -0.797 |  |  |
| 38 | Zhenxi Shen | alpine meadow | 101°19'12"°E | 37°37'12"N | 1998 | N15 | 0.199 | 0.278 | -0.593 |
| 39 | Zhenxi Shen | alpine meadow | 101°19'12"E | 37°37'12"N | 1998 | N30 | -0.097 | 0.103 | -0.847 |
| 40 | Zhenxi Shen | alpine meadow | 101°19'12"°E | 37°37'12"N | 1999 | N15 | 0.216 | 0.487 | -0.792 |
| 41 | Zhenxi Shen | alpine meadow | 101°19'12"E | 37°37'12"N | 1999 | N30 | 0.445 | 0.824 | -1.017 |
| 42 | Zhenxi Shen | alpine meadow | 101°19'12"°E | 37°37'12"N | 2000 | N15 | 0.298 | 0.631 | -0.166 |
| 43 | Zhenxi Shen | alpine meadow | 101°19'12"E | 37°37'12"N | 2000 | N30 | 0.239 | 0.553 | -0.206 |
| 44 | Jieqi Zhang | alpine meadow | 101°52'48"°E | 33°58'11.99"N | 2009 | N11.67 | 0.269 |  |  |
| 45 | Jieqi Zhang | alpine meadow | 101°52'48"°E | 33°58'11.99"N | 2009 | N23.34 | 0.315 |  |  |
| 46 | Jieqi Zhang | alpine meadow | 101°52'48"°E | 33°58'11.99"N | 2009 | N35.01 | 0.288 |  |  |
| 47 | Wei Wang | alpine meadow | 97°18'°E | 33°24'28"N | 2009 | N5 | 0.47 |  |  |
| 48 | Wei Wang | alpine meadow | 97°18'°E | 33°24'28"N | 2009 | N10 | 0.833 |  |  |
| 49 | Wei Wang | alpine meadow | 97°18'°E | 33°24'28"N | 2009 | N15 | 0.734 |  |  |
| 50 | Wei Wang | alpine meadow | 97°18'°E | 33°24'28"N | 2009 | N20 | 0.678 |  |  |
| 51 | Wei Wang | alpine meadow | 97°18'°E | 33°24'28"N | 2009 | N30 | 0.338 |  |  |
| 52 | Wei Wang | alpine meadow | 97°18'°E | 33°24'28"N | 2009 | N35 | 0.44 |  |  |
| 53 | Wei Wang | alpine meadow | 97°18'°E | 33°24'28"N | 2009 | N40 | 0.573 |  |  |
| 54 | Wei Wang | alpine meadow | 97°18'°E | 33°24'28"N | 2009 | N45 | 0.247 |  |  |
| 55 | Wei Wang | alpine meadow | 97°18'°E | 33°24'28"N | 2009 | N5 | 0.337 |  |  |
| 56 | Wei Wang | alpine meadow | 97°18'°E | 33°24'28"N | 2009 | N10 | 0.057 |  |  |
| 57 | Wei Wang | alpine meadow | 97°18'°E | 33°24'28"N | 2009 | N15 | 0.505 |  |  |
| 58 | Wei Wang | alpine meadow | 97°18'°E | 33°24'28"N | 2009 | N20 | 0.685 |  |  |
| 59 | Wei Wang | alpine meadow | 97°18'°E | 33°24'28"N | 2009 | N25 | 1.01 |  |  |
| 60 | Wei Wang | alpine meadow | 97°18'°E | 33°24'28"N | 2009 | N30 | 0.314 |  |  |
| 61 | Wei Wang | alpine meadow | 97°18'°E | 33°24'28"N | 2009 | N35 | 0.339 |  |  |
| 62 | Wei Wang | alpine meadow | 97°18'°E | 33°24'28"N | 2009 | N40 | 0.241 |  |  |
| 63 | Wei Wang | alpine meadow | 97°18'°E | 33°24'28"N | 2009 | N45 | 0.125 |  |  |
| 64 | Wei Wang | alpine meadow | 97°18'°E | 33°24'28"N | 2009 | N5 | 0.393 |  |  |
| 65 | Wei Wang | alpine meadow | 97°18'°E | 33°24'28"N | 2009 | N10 | 0.678 |  |  |
| 66 | Wei Wang | alpine meadow | 97°18'°E | 33°24'28"N | 2009 | N15 | 0.907 |  |  |
| 67 | Wei Wang | alpine meadow | 97°18'°E | 33°24'28"N | 2009 | N25 | 1.092 |  |  |
| 68 | Wei Wang | alpine meadow | 97°18'°E | 33°24'28"N | 2009 | N30 | 0.487 |  |  |
| 69 | Wei Wang | alpine meadow | 97°18'°E | 33°24'28"N | 2009 | N35 | 0.425 |  |  |
| 70 | Wei Wang | alpine meadow | 97°18'°E | 33°24'28"N | 2009 | N40 | 0.271 |  |  |
| 71 | Wei Wang | alpine meadow | 97°18'°E | 33°24'28"N | 2009 | N45 | 0.166 |  |  |
| 72 | Yongfei Bai | Eurasia steppe | 116°42´E | 43°38´N | 2000 | N0 | 0.022 |  |  |
| 73 | Yongfei Bai | Eurasia steppe | 116°42´E | 43°38´N | 2000 | N1.75 | 0.022 |  |  |
| 74 | Yongfei Bai | Eurasia steppe | 116°42´E | 43°38´N | 2000 | N5.25 | 0.16 |  |  |
| 75 | Yongfei Bai | Eurasia steppe | 116°42´E | 43°38´N | 2000 | N10.5 | 0.197 |  |  |
| 76 | Yongfei Bai | Eurasia steppe | 116°42´E | 43°38´N | 2000 | N17.5 | 0.142 |  |  |
| 77 | Yongfei Bai | Eurasia steppe | 116°42´E | 43°38´N | 2000 | N28 | 0.249 |  |  |
| 78 | Yongfei Bai | Eurasia steppe | 116°42´E | 43°38´N | 2001 | N0 | -0.234 |  |  |
| 79 | Yongfei Bai | Eurasia steppe | 116°42´E | 43°38´N | 2001 | N1.75 | -0.179 |  |  |
| 80 | Yongfei Bai | Eurasia steppe | 116°42´E | 43°38´N | 2001 | N5.25 | 0.306 |  |  |
| 81 | Yongfei Bai | Eurasia steppe | 116°42´E | 43°38´N | 2001 | N10.5 | 0.113 |  |  |
| 82 | Yongfei Bai | Eurasia steppe | 116°42´E | 43°38´N | 2001 | N17.5 | 0.238 |  |  |
| 83 | Yongfei Bai | Eurasia steppe | 116°42´E | 43°38´N | 2001 | N28 | 0.273 |  |  |
| 84 | Yongfei Bai | Eurasia steppe | 116°42´E | 43°38´N | 2002 | N0 | -0.034 |  |  |
| 85 | Yongfei Bai | Eurasia steppe | 116°42´E | 43°38´N | 2002 | N1.75 | 0.225 |  |  |
| 86 | Yongfei Bai | Eurasia steppe | 116°42´E | 43°38´N | 2002 | N5.25 | 0.416 |  |  |
| 87 | Yongfei Bai | Eurasia steppe | 116°42´E | 43°38´N | 2002 | N10.5 | 0.682 |  |  |
| 88 | Yongfei Bai | Eurasia steppe | 116°42´E | 43°38´N | 2002 | N17.5 | 0.642 |  |  |
| 89 | Yongfei Bai | Eurasia steppe | 116°42´E | 43°38´N | 2002 | N28 | 0.642 |  |  |
| 90 | Yongfei Bai | Eurasia steppe | 116°42´E | 43°38´N | 2003 | N0 | -0.116 |  |  |
| 91 | Yongfei Bai | Eurasia steppe | 116°42´E | 43°38´N | 2003 | N1.75 | 0.577 |  |  |
| 92 | Yongfei Bai | Eurasia steppe | 116°42´E | 43°38´N | 2003 | N5.25 | 0.739 |  |  |
| 93 | Yongfei Bai | Eurasia steppe | 116°42´E | 43°38´N | 2000 | N0 | -0.087 |  |  |
| 94 | Yongfei Bai | Eurasia steppe | 116°42´E | 43°38´N | 2000 | N1.75 | 0.054 |  |  |
| 95 | Yongfei Bai | Eurasia steppe | 116°42´E | 43°38´N | 2000 | N5.25 | -0.118 |  |  |
| 96 | Yongfei Bai | Eurasia steppe | 116°42´E | 43°38´N | 2000 | N10.5 | -0.057 |  |  |
| 97 | Yongfei Bai | Eurasia steppe | 116°42´E | 43°38´N | 2000 | N17.5 | 0 |  |  |
| 98 | Yongfei Bai | Eurasia steppe | 116°42´E | 43°38´N | 2000 | N28 | 0.054 |  |  |
| 99 | Yongfei Bai | Eurasia steppe | 116°42´E | 43°38´N | 2001 | N0 | 0 |  |  |
| 100 | Yongfei Bai | Eurasia steppe | 116°42´E | 43°38´N | 2001 | N1.75 | 0.09 |  |  |
| 101 | Yongfei Bai | Eurasia steppe | 116°42´E | 43°38´N | 2001 | N5.25 | 0.374 |  |  |
| 102 | Yongfei Bai | Eurasia steppe | 116°42´E | 43°38´N | 2001 | N10.5 | 0.219 |  |  |
| 103 | Yongfei Bai | Eurasia steppe | 116°42´E | 43°38´N | 2001 | N17.5 | 0.449 |  |  |
| 104 | Yongfei Bai | Eurasia steppe | 116°42´E | 43°38´N | 2001 | N28 | 0.306 |  |  |
| 105 | Yongfei Bai | Eurasia steppe | 116°42´E | 43°38´N | 2002 | N0 | 0.022 |  |  |
| 106 | Yongfei Bai | Eurasia steppe | 116°42´E | 43°38´N | 2002 | N1.75 | 0.175 |  |  |
| 107 | Yongfei Bai | Eurasia steppe | 116°42´E | 43°38´N | 2002 | N5.25 | 0.332 |  |  |
| 108 | Yongfei Bai | Eurasia steppe | 116°42´E | 43°38´N | 2002 | N10.5 | 0.379 |  |  |
| 109 | Yongfei Bai | Eurasia steppe | 116°42´E | 43°38´N | 2002 | N17.5 | 0.46 |  |  |
| 110 | Yongfei Bai | Eurasia steppe | 116°42´E | 43°38´N | 2002 | N28 | 0.481 |  |  |
| 111 | Yongfei Bai | Eurasia steppe | 116°42´E | 43°38´N | 2003 | N0 | -0.215 |  |  |
| 112 | Yongfei Bai | Eurasia steppe | 116°42´E | 43°38´N | 2003 | N1.75 | -0.095 |  |  |
| 113 | Yongfei Bai | Eurasia steppe | 116°42´E | 43°38´N | 2003 | N5.25 | 0.077 |  |  |
| 114 | Yongfei Bai | Eurasia steppe | 116°42´E | 43°38´N | 2003 | N10.5 | 0.167 |  |  |
| 115 | Yongfei Bai | Eurasia steppe | 116°42´E | 43°38´N | 2003 | N17.5 | 0.268 |  |  |
| 116 | Yongfei Bai | Eurasia steppe | 116°42´E | 43°38´N | 2003 | N28 | 0.186 |  |  |
| 117 | Li K | alpine grassland | 83°42.5′E | 42°53.1′N | 2009 | N1 | 0.205 |  |  |
| 118 | Li K | alpine grassland | 83°42.5′E | 42°53.1′N | 2009 | N3 | 0.276 |  |  |
| 119 | Li K | alpine grassland | 83°42.5′E | 42°53.1′N | 2009 | N9 | 0.52 |  |  |
| 120 | Li K | alpine grassland | 83°42.5′E | 42°53.1′N | 2010 | N1 | 0.025 |  |  |
| 121 | Li K | alpine grassland | 83°42.5′E | 42°53.1′N | 2010 | N3 | 0.165 |  |  |
| 122 | Li K | alpine grassland | 83°42.5′E | 42°53.1′N | 2010 | N9 | 0.249 |  |  |
| 123 | Li K | alpine grassland | 83°42.5′E | 42°53.1′N | 2011 | N1 | 0.256 |  |  |
| 124 | Li K | alpine grassland | 83°42.5′E | 42°53.1′N | 2011 | N3 | 0.368 |  |  |
| 125 | Li K | alpine grassland | 83°42.5′E | 42°53.1′N | 2011 | N9 | 0.154 |  |  |
| 126 | Li K | alpine grassland | 83°42.5′E | 42°53.1′N | 2012 | N1 | 0 |  |  |
| 127 | Li K | alpine grassland | 83°42.5′E | 42°53.1′N | 2012 | N3 | 0.125 |  |  |
| 128 | Li K | alpine grassland | 83°42.5′E | 42°53.1′N | 2012 | N9 | 0.182 |  |  |
| 129 | Li K | alpine grassland | 83°42.5′E | 42°53.1′N | 2009 | N30W | 0.316 |  |  |
| 130 | Li K | alpine grassland | 83°42.5′E | 42°53.1′N | 2010 | N30W | 0.413 |  |  |
| 131 | Li K | alpine grassland | 83°42.5′E | 42°53.1′N | 2011 | N30W | 0.175 |  |  |
| 132 | Yuchun Qi | Typical steppe | 116°40′31"E | 43°33´3"N | 2009 | N10 | -0.236 |  |  |
| 133 | Yuchun Qi | Typical steppe | 116°40′31"E | 43°33´3"N | 2009 | N10 | -0.423 |  |  |
| 134 | Yongfei Bai | Typical steppe | 116°40′31"E | 43°33´3"N | 2010 | N10 | 0.393 |  |  |
| 135 | Yongfei Bai | Typical steppe | 116°40′31"E | 43°33´3"N | 2010 | N10 | 0.174 |  |  |
| 136 | Hongyu Guo | alpine meadow | 97°18´00"E | 32°24′30"N | 2013 | N30 | 0.611 |  |  |
| 137 | Xiaojuan Xin | alpine meadow | 102°53´E | 34°55´N | 2010 | N5 | 0.211 | 0.26 | 1.259 |
| 138 | Xiaojuan Xin | alpine meadow | 102°53´E | 34°55´N | 2010 | N10 | 0.439 | 0.612 | 1.332 |
| 139 | Xiaojuan Xin | alpine meadow | 102°53´E | 34°55´N | 2010 | N15 | 0.348 | 0.471 | 1.276 |
| 140 | Xiaojuan Xin | alpine meadow | 102°53´E | 34°55´N | 2010 | N5P5 | -0.125 | -0.081 | -0.113 |
| 141 | Xiaojuan Xin | alpine meadow | 102°53´E | 34°55´N | 2010 | N10P10 | 0.487 | 0.908 | -0.306 |
| 142 | Xiaojuan Xin | alpine meadow | 102°53´E | 34°55´N | 2010 | N15P15 | 0.613 | 0.889 | -0.108 |
| 143 | L. Song | typical steppe | 116°17´E | 42°02´N | 2005 | N6 | 0.348 | 0.511 | 0.231 |
| 144 | L. Song | typical steppe | 116°17´E | 42°02´N | 2005 | N12 | 0.439 | 0.56 | 0.355 |
| 145 | L. Song | typical steppe | 116°17´E | 42°02´N | 2005 | N24 | 0.703 | 0.981 | 0.477 |
| 146 | L. Song | typical steppe | 116°17´E | 42°02´N | 2005 | N48 | 0.757 | 1.012 | 0.554 |
| 147 | L. Song | typical steppe | 116°17´E | 42°02´N | 2006 | N3 | -0.186 | 0.105 | -0.357 |
| 148 | L. Song | typical steppe | 116°17´E | 42°02´N | 2006 | N6 | 0.148 | 0.575 | -0.138 |
| 149 | L. Song | typical steppe | 116°17´E | 42°02´N | 2006 | N12 | 0.451 | 1.329 | -0.611 |
| 150 | L. Song | typical steppe | 116°17´E | 42°02´N | 2006 | N24 | 0.621 | 1.492 | -0.42 |
| 151 | L. Song | typical steppe | 116°17´E | 42°02´N | 2006 | N48 | 0.872 | 1.977 | -1.946 |
| 152 | L. Song | typical steppe | 116°17´E | 42°02´N | 2008 | N3 | 0.253 | 0.475 | -0.051 |
| 153 | L. Song | typical steppe | 116°17´E | 42°02´N | 2008 | N6 | 0.386 | 0.693 | -0.092 |
| 154 | L. Song | typical steppe | 116°17´E | 42°02´N | 2008 | N12 | 0.483 | 1.039 | -1.05 |
| 155 | L. Song | typical steppe | 116°17´E | 42°02´N | 2008 | N24 | 0.815 | 1.418 | -1.247 |
| 156 | L. Song | typical steppe | 116°17´E | 42°02´N | 2008 | N48 | 0.85 | 1.48 | -1.743 |
| 157 | L. Song | typical steppe | 116°17´E | 42°02´N | 2009 | N3 | 0.312 | 0.669 | -0.251 |
| 158 | L. Song | typical steppe | 116°17´E | 42°02´N | 2009 | N6 | 0.304 | 0.847 | -0.981 |
| 159 | L. Song | typical steppe | 116°17´E | 42°02´N | 2009 | N12 | 0.387 | 1.016 | -1.712 |
| 160 | L. Song | typical steppe | 116°17´E | 42°02´N | 2009 | N24 | 0.862 | 1.53 | -2.197 |
| 161 | L. Song | typical steppe | 116°17´E | 42°02´N | 2009 | N48 | 0.672 | 1.35 | -2.89 |
| 162 | L. Song | typical steppe | 116°17´E | 42°02´N | 2010 | N3 | 0.486 | 0.817 | -0.449 |
| 163 | L. Song | typical steppe | 116°17´E | 42°02´N | 2010 | N6 | 0.688 | 1.007 | -0.187 |
| 164 | Yu Qi | Typical steppe | 115°29'10"E | 42°06'44"N | 2011 | N2 | -0.296 |  |  |
| 165 | Yu Qi | Typical steppe | 115°29'10"E | 42°06'44"N | 2011 | N5 | 0.029 |  |  |
| 166 | Yu Qi | Typical steppe | 115°29'10"E | 42°06'44"N | 2011 | N10 | -0.23 |  |  |
| 167 | Yu Qi | Typical steppe | 115°29'10"E | 42°06'44"N | 2011 | N25 | -0.08 |  |  |
| 168 | Changting Wang | alpine meadow | 100°29'—99°54'E | 34°30'—33°34'N | 2009 | N12 | 0.259 | 0.125 | 0.29 |
| 169 | Changting Wang | alpine meadow | 100°29'—99°54'E | 34°30'—33°34'N | 2009 | N20 | 0.488 | 0.924 | 0.347 |
| 170 | Changting Wang | alpine meadow | 100°29'—99°54'E | 34°30'—33°34'N | 2009 | N32 | 0.523 | 1.083 | 0.322 |
| 171 | Changting Wang | alpine meadow | 100°29'—99°54'E | 34°30'—33°34'N | 2009 | N40 | 0.11 | 0.652 | -0.081 |
| 172 | Yu Qi | Typical steppe | 115°29´E | 42°06´N | 2009 | N30 | 0.318 |  |  |
| 173 | Yu Qi | Typical steppe | 115°29´E | 42°06´N | 2009 | N60 | 0.416 |  |  |
| 174 | Lujun Li | Typical steppe | 122°21´E | 42°58´N | 2008 | N2.5 | 0.408 |  |  |
| 175 | Lujun Li | Typical steppe | 122°21´E | 42°58´N | 2008 | N5 | 0.543 |  |  |
| 176 | Lujun Li | Typical steppe | 122°21´E | 42°58´N | 2008 | N7.5 | 0.939 |  |  |
| 177 | Lujun Li | Typical steppe | 122°21´E | 42°58´N | 2008 | N10 | 0.84 |  |  |
| 178 | Lujun Li | Typical steppe | 122°21´E | 42°58´N | 2008 | N30 | 0.511 |  |  |
| 179 | Zhanyuan Yu | Typical steppe | 122°21´E | 42°58´N | 2004 | N10 | 0.947 |  |  |
| 180 | Zhanyuan Yu | Typical steppe | 122°21´E | 42°58´N | 2004 | N10W | 1.03 |  |  |
| 181 | Zhanyuan Yu | Typical steppe | 122°21´E | 42°58´N | 2004 | N10P | 0.902 |  |  |
| 182 | Xing He | Meadow steppe | 120°7´E | 49°21´N | 2011 | N10 | 0.223 |  |  |
| 183 | Xing He | Meadow steppe | 120°7´E | 49°21´N | 2011 | N2.5 | 0.134 |  |  |
| 184 | Xing He | Meadow steppe | 120°7´E | 49°21´N | 2011 | N5 | -0.19 |  |  |
| 185 | Xing He | Meadow steppe | 120°7´E | 49°21´N | 2011 | N10P | 0.166 |  |  |
| 186 | Xing He | Meadow steppe | 120°7´E | 49°21´N | 2012 | N10 | 0.368 |  |  |
| 187 | Xing He | Meadow steppe | 120°7´E | 49°21´N | 2012 | N2.5 | 0.247 |  |  |
| 188 | Xing He | Meadow steppe | 120°7´E | 49°21´N | 2012 | N5 | 0.178 |  |  |
| 189 | Xing He | Meadow steppe | 120°7´E | 49°21´N | 2012 | N10P | 0.423 |  |  |
| 190 | Xing He | Meadow steppe | 120°7´E | 49°21´N | 2013 | N10 | 0.273 |  |  |
| 191 | Xing He | Meadow steppe | 120°7´E | 49°21´N | 2013 | N2.5 | 0.101 |  |  |
| 192 | Xing He | Meadow steppe | 120°7´E | 49°21´N | 2013 | N5 | 0.164 |  |  |
| 193 | Xing He | Meadow steppe | 120°7´E | 49°21´N | 2013 | N10P | 0.611 |  |  |
| 194 | Xing He | Typical steppe | 116°28´56.8´´E | 44°10´02.4´´N | 2011 | N10 | 0.03 |  |  |
| 195 | Xing He | Typical steppe | 116°28´56.8´´E | 44°10´02.4´´N | 2011 | N2.5 | -0.128 |  |  |
| 196 | Xing He | Typical steppe | 116°28´56.8´´E | 44°10´02.4´´N | 2011 | N5 | 0.205 |  |  |
| 197 | Xing He | Typical steppe | 116°28´56.8´´E | 44°10´02.4´´N | 2011 | N10P | -0.009 |  |  |
| 198 | Xing He | Typical steppe | 116°28´56.8´´E | 44°10´02.4´´N | 2012 | N10 | 0.201 |  |  |
| 199 | Xing He | Typical steppe | 116°28´56.8´´E | 44°10´02.4´´N | 2012 | N2.5 | 0.03 |  |  |
| 200 | Xing He | Typical steppe | 116°28´56.8´´E | 44°10´02.4´´N | 2012 | N5 | 0.064 |  |  |
| 201 | Xing He | Typical steppe | 116°28´56.8´´E | 44°10´02.4´´N | 2012 | N10P | 0.274 |  |  |
| 202 | Xing He | Typical steppe | 116°28´56.8´´E | 44°10´02.4´´N | 2013 | N10 | 0.384 |  |  |
| 203 | Xing He | Typical steppe | 116°28´56.8´´E | 44°10´02.4´´N | 2013 | N2.5 | 0.115 |  |  |
| 204 | Xing He | Typical steppe | 116°28´56.8´´E | 44°10´02.4´´N | 2013 | N5 | 0.257 |  |  |
| 205 | Xing He | Typical steppe | 116°28´56.8´´E | 44°10´02.4´´N | 2013 | N10P | 0.651 |  |  |
| 206 | Xing He | desert steppe | 111°13´28.2´´E | 41°18´07.9´´N | 2011 | N10 | 0.167 |  |  |
| 207 | Xing He | desert steppe | 111°13´28.2´´E | 41°18´07.9´´N | 2011 | N2.5 | -0.069 |  |  |
| 208 | Xing He | desert steppe | 111°13´28.2´´E | 41°18´07.9´´N | 2011 | N5 | 0.15 |  |  |
| 209 | Xing He | desert steppe | 111°13´28.2´´E | 41°18´07.9´´N | 2011 | N10P | 0.076 |  |  |
| 210 | Xing He | desert steppe | 111°13´28.2´´E | 41°18´07.9´´N | 2012 | N10 | 0.512 |  |  |
| 211 | Xing He | desert steppe | 111°13´28.2´´E | 41°18´07.9´´N | 2012 | N2.5 | 0.204 |  |  |
| 212 | Xing He | desert steppe | 111°13´28.2´´E | 41°18´07.9´´N | 2012 | N5 | 0.289 |  |  |
| 213 | Xing He | desert steppe | 111°13´28.2´´E | 41°18´07.9´´N | 2012 | N10P | 0.674 |  |  |
| 214 | Xing He | desert steppe | 111°13´28.2´´E | 41°18´07.9´´N | 2013 | N10 | 0.555 |  |  |
| 215 | Xing He | desert steppe | 111°13´28.2´´E | 41°18´07.9´´N | 2013 | N2.5 | 0.364 |  |  |
| 216 | Xing He | desert steppe | 111°13´28.2´´E | 41°18´07.9´´N | 2013 | N5 | 0.431 |  |  |
| 217 | Xing He | desert steppe | 111°13´28.2´´E | 41°18´07.9´´N | 2013 | N10P | 0.467 |  |  |
| 218 | Xue Bai | Typical steppe | 116°40´30´´-116°40´50´´E | 43°32´45´´-43°33´10´´N | 2011 | N10.5 | 0.133 |  |  |
| 219 | Xue Bai | Typical steppe | 116°40´30´´-116°40´50´´E | 43°32´45´´-43°33´10´´N | 2011 | N10.5P | 0.422 |  |  |
| 220 | Xue Bai | Typical steppe | 116°40´30´´-116°40´50´´E | 43°32´45´´-43°33´10´´N | 2012 | N10.5 | 0.278 |  |  |
| 221 | Xue Bai | Typical steppe | 116°40´30´´-116°40´50´´E | 43°32´45´´-43°33´10´´N | 2012 | N10.5P | 0.465 |  |  |
| 222 | Qingmin Pan | Typical steppe | 116°40´30´´-116°40´50´´E | 43°32´45´´-43°33´10´´N | 2001 | N5.25 | 0.34 |  |  |
| 223 | Qingmin Pan | Typical steppe | 116°40´30´´-116°40´50´´E | 43°32´45´´-43°33´10´´N | 2001 | N10.5 | 0.601 |  |  |
| 224 | Qingmin Pan | Typical steppe | 116°40´30´´-116°40´50´´E | 43°32´45´´-43°33´10´´N | 2001 | N17.5 | 0.964 |  |  |
| 225 | Qingmin Pan | Typical steppe | 116°40´30´´-116°40´50´´E | 43°32´45´´-43°33´10´´N | 2001 | N28 | 1.081 |  |  |
| 226 | Haiyang Ren | Typical steppe | 116°17′E | 42°04′N | 2007 | N10 | 0.045 | 0.84 | 0.391 |
| 227 | Haiyang Ren | Typical steppe | 116°17′E | 42°04′N | 2007 | N10W | 0.214 | 0.452 | -0.726 |
| 228 | Haiyang Ren | Typical steppe | 116°17′E | 42°04′N | 2008 | N10 | 0.265 | 1.081 | 0.065 |
| 229 | Haiyang Ren | Typical steppe | 116°17′E | 42°04′N | 2008 | N10W | 0.348 | 0.65 | -0.565 |
| 230 | Jieqiong Su | desert steppe | 104°35´E | 37°25´N | 2007 | N0 | 0.052 |  |  |
| 231 | Jieqiong Su | desert steppe | 104°35´E | 37°25´N | 2007 | N3.5 | 0.117 |  |  |
| 232 | Jieqiong Su | desert steppe | 104°35´E | 37°25´N | 2007 | N7 | 0.379 |  |  |
| 233 | Jieqiong Su | desert steppe | 104°35´E | 37°25´N | 2007 | N14 | 0.605 |  |  |
| 234 | Jieqiong Su | desert steppe | 104°35´E | 37°25´N | 2008 | N0 | -0.043 |  |  |
| 235 | Jieqiong Su | desert steppe | 104°35´E | 37°25´N | 2008 | N3.5 | -0.271 |  |  |
| 236 | Jieqiong Su | desert steppe | 104°35´E | 37°25´N | 2008 | N7 | -0.427 |  |  |
| 237 | Jieqiong Su | desert steppe | 104°35´E | 37°25´N | 2009 | N0 | -0.02 |  |  |
| 238 | Jieqiong Su | desert steppe | 104°35´E | 37°25´N | 2009 | N3.5 | -0.174 |  |  |
| 239 | Jieqiong Su | desert steppe | 104°35´E | 37°25´N | 2010 | N0 | 0 |  |  |
| 240 | Jieqiong Su | desert steppe | 104°35´E | 37°25´N | 2010 | N3.5 | -0.182 |  |  |
| 241 | Jieqiong Su | desert steppe | 104°35´E | 37°25´N | 2010 | N7 | -0.588 |  |  |
| 242 | Ying Fang | Typical steppe | 116°17´E | 42°02´N | 2010 | N2 | 0.173 |  |  |
| 243 | Ying Fang | Typical steppe | 116°17´E | 42°02´N | 2010 | N8 | 0.475 |  |  |
| 244 | Ying Fang | Typical steppe | 116°17´E | 42°02´N | 2010 | N16 | 0.525 |  |  |
| 245 | Ying Fang | Typical steppe | 116°17´E | 42°02´N | 2010 | N32 | 0.286 |  |  |
| 246 | Xiaotao Lü | Typical steppe | 116°42´E | 43°38´N | 2007 | N17.5 | 0.118 |  |  |
| 247 | Xiaotao Lü | Typical steppe | 116°42´E | 43°38´N | 2007 | N17.5W | 0.004 |  |  |
| 248 | Xiaotao Lü | Typical steppe | 116°42´E | 43°38´N | 2008 | N17.5 | 0.249 |  |  |
| 249 | Xiaotao Lü | Typical steppe | 116°42´E | 43°38´N | 2008 | N17.5W | -0.061 |  |  |
| 250 | Wang Changhui | Arid and semi arid grassland | 116°17′E | 42°02′N | 2009 | N10 | 0.496 |  |  |
| 251 | Wang Changhui | Arid and semi arid grassland | 116°17′E | 42°02′N | 2009 | N10P | -0.613 |  |  |
| 252 | Kaihui Li | alpine meadow | 83°42.5′E | 42°53.1′N | 2009 | N1 | 0.201 | 0.452 | 0 |
| 253 | Kaihui Li | alpine meadow | 83°42.5′E | 42°53.1′N | 2009 | N3 | 0.288 | 0.693 | -0.201 |
| 254 | Kaihui Li | alpine meadow | 83°42.5′E | 42°53.1′N | 2009 | N9 | 0.544 | 0.999 | 0.087 |
| 255 | Kaihui Li | alpine meadow | 83°42.5′E | 42°53.1′N | 2010 | N1 | 0.031 | 0.167 | 0 |
| 256 | Kaihui Li | alpine meadow | 83°42.5′E | 42°53.1′N | 2010 | N3 | 0.172 | 0.493 | 0 |
| 257 | Kaihui Li | alpine meadow | 83°42.5′E | 42°53.1′N | 2010 | N9 | 0.248 | 0.78 | -0.118 |
| 258 | Kaihui Li | alpine meadow | 83°42.5′E | 42°53.1′N | 2011 | N1 | 0.253 | 0.145 | 0.38 |
| 259 | Kaihui Li | alpine meadow | 83°42.5′E | 42°53.1′N | 2011 | N3 | 0.377 | 0.739 | -0.425 |
| 260 | Kaihui Li | alpine meadow | 83°42.5′E | 42°53.1′N | 2011 | N9 | 0.157 | 0.56 | -0.86 |
| 261 | Kaihui Li | alpine meadow | 83°42.5′E | 42°53.1′N | 2012 | N1 | 0 | 0 | 0 |
| 262 | Kaihui Li | alpine meadow | 83°42.5′E | 42°53.1′N | 2012 | N3 | 0.113 | 0.234 | -0.916 |
| 263 | Kaihui Li | alpine meadow | 83°42.5′E | 42°53.1′N | 2012 | N9 | 0.182 | 0.314 | -0.916 |
| 264 | Kaihui Li | alpine meadow | 83°42.5′E | 42°53.1′N | 2009 | N3W | 0.337 | 0.329 | 0.319 |
| 265 | Kaihui Li | alpine meadow | 83°42.5′E | 42°53.1′N | 2010 | N3W | 0.395 | 0.511 | 0.236 |
| 266 | Kaihui Li | alpine meadow | 83°42.5′E | 42°53.1′N | 2011 | N3W | 0.226 | 0.27 | -0.288 |
| 267 | Kaihui Li | alpine meadow | 83°42.5′E | 42°53.1′N | 2012 | N3W | 0.387 | 0.478 | -0.223 |
| 268 | Yu Zhanyuan | Typical steppe | 122°2l´E | 42°58´N | 2005 | N20P | 0.719 | 1.984 | -1.22 |
| 269 | Ning Zong | alpine meadow | 91°05´E | 30°51´N | 2010 | N10 | 0.089 |  |  |
| 270 | Ning Zong | alpine meadow | 91°05´E | 30°51´N | 2010 | N20 | 0.345 |  |  |
| 271 | Ning Zong | alpine meadow | 91°05´E | 30°51´N | 2010 | N40 | 0.595 |  |  |
| 272 | Wei Fan | Typical steppe | 116°17′20″E | 42°02′29″N | 2009 | N5 | 0.469 |  |  |
| 273 | Wei Fan | Typical steppe | 116°17′20″E | 42°02′29″N | 2009 | N15 | 0.206 |  |  |
| 274 | Wenjiao Li | Meadow steppe | 119°35´—119°41´E | 48°27´—48°35´N | 2013 | N1.5 | 0.085 | 0.212 | -0.08 |
| 275 | Wenjiao Li | Meadow steppe | 119°35´—119°41´E | 48°27´—48°35´N | 2013 | N3 | 0.306 | 0.487 | 0.056 |
| 276 | Wenjiao Li | Meadow steppe | 119°35´—119°41´E | 48°27´—48°35´N | 2013 | N5 | 0.595 | 0.87 | 0.157 |
| 277 | Wenjiao Li | Meadow steppe | 119°35´—119°41´E | 48°27´—48°35´N | 2013 | N10 | 0.974 | 1.332 | 0.314 |
| 278 | Wenjiao Li | Meadow steppe | 119°35´—119°41´E | 48°27´—48°35´N | 2013 | N15 | 0.956 | 1.364 | 0.121 |
| 279 | Wenjiao Li | Meadow steppe | 119°35´—119°41´E | 48°27´—48°35´N | 2013 | N20 | 0.965 | 1.42 | -0.076 |
| 280 | Wenjiao Li | Meadow steppe | 119°35´—119°41´E | 48°27´—48°35´N | 2013 | N30 | 0.869 | 1.371 | -0.442 |
| 281 | Wenjiao Li | Meadow steppe | 119°35´—119°41´E | 48°27´—48°35´N | 2013 | N1.5W | -0.008 | 0.326 | -0.354 |
| 282 | Wenjiao Li | Meadow steppe | 119°35´—119°41´E | 48°27´—48°35´N | 2013 | N3W | 0.219 | 0.718 | -0.429 |
| 283 | Wenjiao Li | Meadow steppe | 119°35´—119°41´E | 48°27´—48°35´N | 2013 | N5W | 0.512 | 1.076 | -0.306 |
| 284 | Wenjiao Li | Meadow steppe | 119°35´—119°41´E | 48°27´—48°35´N | 2013 | N10W | 0.732 | 1.406 | -0.508 |
| 285 | Wenjiao Li | Meadow steppe | 119°35´—119°41´E | 48°27´—48°35´N | 2013 | N15W | 1.012 | 1.768 | -0.785 |
| 286 | Wenjiao Li | desert steppe | 111°53´46´´E | 41°47′17″N | 2011 | N17.5 | 0.164 | 1.929 | -0.981 |
| 287 | Wenjiao Li | desert steppe | 111°53´46´´E | 41°47′17″N | 2011 | N17.5W | 0.184 |  |  |
| 288 | Song Gao | desert steppe | 123°44´-123°47´E | 44°40`-44°44`N | 2006 | N10 | 0.304 |  |  |
| 289 | Song Gao | desert steppe | 123°44´-123°47´E | 44°40`-44°44`N | 2007 | N10 | 0.12 |  |  |
| 290 | Song Gao | desert steppe | 123°44´-123°47´E | 44°40`-44°44`N | 2008 | N10 | 0.136 |  |  |
| 291 | Song Gao | desert steppe | 123°44´-123°47´E | 44°40`-44°44`N | 2009 | N10 | 0.089 |  |  |
| 292 | Jieqi Zhang | alpine meadow | 101°53´E | 33°58´N | 2009 | N11.67 | 0.272 |  |  |
| 293 | Jieqi Zhang | alpine meadow | 101°53´E | 33°58´N | 2009 | N23.34 | 0.318 |  |  |
| 294 | Jieqi Zhang | alpine meadow | 101°53´E | 33°58´N | 2009 | N35.01 | 0.293 |  |  |
| 295 | Dan He | Typical steppe | 115°51′–115°49´E | 41°35´N-42°10´N | 2008 | N2.5 | 0.186 |  |  |
| 296 | Dan He | Typical steppe | 115°51′–115°49´E | 41°35´N-42°10´N | 2008 | N5 | 0.395 |  |  |
| 297 | Dan He | Typical steppe | 115°51′–115°49´E | 41°35´N-42°10´N | 2008 | N7.5 | 0.392 |  |  |
| 298 | Dan He | Typical steppe | 115°51′–115°49´E | 41°35´N-42°10´N | 2008 | N10 | 0.425 |  |  |
| 299 | Yandong Zhang |  | 103°E | 26°N | 2000 | N5 | 0.254 |  |  |
| 300 | Yandong Zhang |  | 103°E | 26°N | 2000 | N15 | 0.551 |  |  |
| 301 | Yandong Zhang |  | 103°E | 26°N | 2000 | N25 | 0.494 |  |  |
| 302 | Tonghui Zhang | Sandy meadow | 120°42´E | 42°55´N | 2001 | N15 | 0.296 |  |  |
| 303 | Tonghui Zhang | Sandy meadow | 120°42´E | 42°55´N | 2001 | N37.5 | 0.758 |  |  |
| 304 | Tonghui Zhang | Sandy meadow | 120°42´E | 42°55´N | 2001 | N60 | 0.792 |  |  |
| 305 | Huaping Zheng | Sandy meadow | 102°09´30.3"E | 34°0039.0"´N | 2006 | N25 | 0.409 |  |  |
| 306 | Huaping Zheng | Sandy meadow | 102°09´30.3"E | 34°0039.0"´N | 2006 | N25P | 0.123 |  |  |
| 307 | Birong Liu | Typical steppe | 116°17′E | 42°02´N | 2011 | N10 | 0.536 |  |  |
| 308 | Birong Liu | Typical steppe | 116°17′E | 42°02´N | 2012 | N10 | 0.803 |  |  |
| 309 | Birong Liu | Typical steppe | 116°17′E | 42°02´N | 2013 | N10 | 0.884 |  |  |
| 310 | Birong Liu | Typical steppe | 116°14′E | 43°13′N | 2011 | N2 | 0.048 |  |  |
| 311 | Birong Liu | Typical steppe | 116°14′E | 43°13′N | 2011 | N10 | 0.307 |  |  |
| 312 | Birong Liu | Typical steppe | 116°14′E | 43°13′N | 2011 | N20 | 0.449 |  |  |
| 313 | Birong Liu | Typical steppe | 116°14′E | 43°13′N | 2011 | N50 | 0.422 |  |  |
| 314 | Birong Liu | Typical steppe | 116°14′E | 43°13′N | 2011 | N2 | 0.137 |  |  |
| 315 | Birong Liu | Typical steppe | 116°14′E | 43°13′N | 2011 | N10 | 0.261 |  |  |
| 316 | Birong Liu | Typical steppe | 116°14′E | 43°13′N | 2011 | N20 | 0.389 |  |  |
| 317 | Birong Liu | Typical steppe | 116°14′E | 43°13′N | 2011 | N50 | 0.577 |  |  |
| 318 | Xiao YingGong | typical steppe | 115°32´-117°12´E | 43°26′–44°29′N | 2008 | N7.5 | -0.122 |  |  |
| 319 | Xiao YingGong | typical steppe | 115°32´-117°12´E | 43°26′–44°29′N | 2008 | N7.5W | -0.207 |  |  |
| 320 | Hongwei Wan | typical steppe | 115°32´-117°12´E | 43°26′–44°29′N | 2005 | N5.25 | 0.078 |  |  |
| 321 | Hongwei Wan | typical steppe | 115°32´-117°12´E | 43°26′–44°29′N | 2005 | N10.5 | 0.146 |  |  |
| 322 | Hongwei Wan | typical steppe | 115°32´-117°12´E | 43°26′–44°29′N | 2005 | N17.5 | 0.386 |  |  |
| 323 | Hongwei Wan | typical steppe | 115°32´-117°12´E | 43°26′–44°29′N | 2005 | N28 | 0.177 |  |  |
| 324 | Liqing Qiao | Typical steppe | 116°04´E-117°05´E | 43°26´N-44°08´N | 2012 | N10 | 0.15 |  |  |
| 325 | Liqing Qiao | Typical steppe | 116°04´E-117°05´E | 43°26´N-44°08´N | 2012 | N10W | 0.215 |  |  |
| 326 | Liqing Qiao | Typical steppe | 116°04´E-117°05´E | 43°26´N-44°08´N | 2013 | N10 | 0.265 |  |  |
| 327 | Liqing Qiao | Typical steppe | 116°04´E-117°05´E | 43°26´N-44°08´N | 2013 | N10W | 0.311 |  |  |
| 328 | Xingren Liu | meadow steppe | 119°55´E-119°58´E | 49°19´N-49°20´N | 2008 | N1 | 0.397 |  |  |
| 329 | Xingren Liu | meadow steppe | 119°55´E-119°58´E | 49°19´N-49°20´N | 2008 | N2 | 0.314 |  |  |
| 330 | Wei Luo | Meadow steppe | 123°44´-123°47´E | 44°40`-44°44`N | 2009 | N10 | 0.247 |  |  |
| 331 | Xiyong Liu | Meadow steppe | 122°55.043´E | 44°34.12´N | 2008 | N5 | 0.115 | 0.127 | 0.153 |
| 332 | Xiyong Liu | Meadow steppe | 122°55.043´E | 44°34.12´N | 2008 | N10 | 0.276 | 0.26 | 0.32 |
| 333 | Xiyong Liu | Meadow steppe | 122°55.043´E | 44°34.12´N | 2008 | N15 | -0.364 | -0.3 | 0.561 |
| 334 | Xiyong Liu | Meadow steppe | 122°55.043´E | 44°34.12´N | 2008 | N20 | -0.396 | -0.315 | 0.421 |
| 335 | Xiyong Liu | Meadow steppe | 122°55.043´E | 44°34.12´N | 2009 | N5 | -0.017 | 0 | 0.375 |
| 336 | Xiyong Liu | Meadow steppe | 122°55.043´E | 44°34.12´N | 2009 | N10 | 0.074 | 0.079 | 0.221 |
| 337 | Xiyong Liu | Meadow steppe | 122°55.043´E | 44°34.12´N | 2009 | N15 | 0.247 | 0.267 | 0.66 |
| 338 | Xiyong Liu | Meadow steppe | 122°55.043´E | 44°34.12´N | 2009 | N20 | 0.167 | 0.193 | 0.965 |
| 339 | Xiyong Liu | Meadow steppe | 122°55.043´E | 44°34.12´N | 2008 | N5 | 0.142 |  |  |
| 340 | Xiyong Liu | Meadow steppe | 122°55.043´E | 44°34.12´N | 2008 | N10 | 0.296 |  |  |
| 341 | Xiyong Liu | Meadow steppe | 122°55.043´E | 44°34.12´N | 2008 | N15 | 0.293 |  |  |
| 342 | Xiyong Liu | Meadow steppe | 122°55.043´E | 44°34.12´N | 2008 | N20 | 0.183 |  |  |
| 343 | Xiyong Liu | Meadow steppe | 122°55.043´E | 44°34.12´N | 2009 | N5 | 0.237 |  |  |
| 344 | Xiyong Liu | Meadow steppe | 122°55.043´E | 44°34.12´N | 2009 | N10 | 0.165 |  |  |
| 345 | Xiyong Liu | Meadow steppe | 122°55.043´E | 44°34.12´N | 2009 | N15 | 0.516 |  |  |
| 346 | Xiyong Liu | Meadow steppe | 122°55.043´E | 44°34.12´N | 2009 | N20 | 0.714 |  |  |
| 347 | Tchister Morrel EBISSA | meadow steppe | 123°31´E | 45°45´N | 2009 | N10 | 0.467 |  |  |
| 348 | Tchister Morrel EBISSA | meadow steppe | 123°31´E | 45°45´N | 2010 | N10 | 0.339 |  |  |
| 349 | Tchister Morrel EBISSA | meadow steppe | 123°31´E | 45°45´N | 2011 | N10 | 0.411 |  |  |
| 350 | Tchister Morrel EBISSA | meadow steppe | 123°31´E | 45°45´N | 2011 | N10 | 0.438 | 0.536 | -0.262 |
| 351 | Zhilong Zhang | alpine meadow | 101°52´E | 33°40´N | 2009 | N5 | 0.075 | 0.195 | 0.021 |
| 352 | Zhilong Zhang | alpine meadow | 101°52´E | 33°40´N | 2009 | N10 | 0.359 | 0.779 | 0.113 |
| 353 | Zhilong Zhang | alpine meadow | 101°52´E | 33°40´N | 2010 | N5 | 0.27 | 0.058 | 0.564 |
| 354 | Zhilong Zhang | alpine meadow | 101°52´E | 33°40´N | 2010 | N10 | 0.37 | 0 | 0.811 |
| 355 | Zhilong Zhang | alpine meadow | 101°52´E | 33°40´N | 2011 | N5 | 0.253 | 0.74 | -0.131 |
| 356 | Zhilong Zhang | alpine meadow | 101°52´E | 33°40´N | 2011 | N10 | 0.314 | 0.957 | -0.297 |
| 357 | Zhilong Zhang | desert steppe | 111°53´41.7´´E | 41°46′43.6″N | 2010 | N10 | -0.013 |  |  |
| 358 | Zhilong Zhang | desert steppe | 111°53´41.7´´E | 41°46′43.6″N | 2011 | N10 | -0.168 |  |  |
| 359 | Yuanheng Li | desert steppe | 111°53´46´´E | 41°47′17″N | 2006 | N10 | 0.035 |  |  |
| 360 | Yuanheng Li | desert steppe | 111°53´46´´E | 41°47′17″N | 2007 | N10 | -0.206 |  |  |
| 361 | Yuanheng Li | desert steppe | 111°53´46´´E | 41°47′17″N | 2008 | N10 | 0.026 |  |  |
| 362 | Yuanheng Li | desert steppe | 111°53´46´´E | 41°47′17″N | 2009 | N10 | 0.082 |  |  |
| 363 | Yuanheng Li | desert steppe | 111°53´46´´E | 41°47′17″N | 2010 | N10 | -0.327 |  |  |
| 364 | Yingzhi Gao | Typical steppe | 116°42´E | 43°38′N | 2006 | N2.5 | 0.026 |  |  |
| 365 | Yingzhi Gao | Typical steppe | 116°42´E | 43°38′N | 2006 | N2.5W | 0.378 |  |  |
| 366 | Dima Chen | Typical steppe | 116°42´E | 43°38′N | 2011 | N1.75 | 0.249 |  |  |
| 367 | Dima Chen | Typical steppe | 116°42´E | 43°38′N | 2011 | N5.25 | 0.388 |  |  |
| 368 | Dima Chen | Typical steppe | 116°42´E | 43°38′N | 2011 | N10.5 | 0.307 |  |  |
| 369 | Dima Chen | Typical steppe | 116°42´E | 43°38′N | 2011 | N17.5 | 0.563 |  |  |
| 370 | Dima Chen | Typical steppe | 116°42´E | 43°38′N | 2011 | N28 | 0.585 |  |  |
| 371 | Dima Chen | Typical steppe | 116°42´E | 43°38′N | 2012 | N1.75 | 0.377 |  |  |
| 372 | Dima Chen | Typical steppe | 116°42´E | 43°38′N | 2012 | N5.25 | 0.174 |  |  |
| 373 | Dima Chen | Typical steppe | 116°42´E | 43°38′N | 2012 | N10.5 | 0.415 |  |  |
| 374 | Dima Chen | Typical steppe | 116°42´E | 43°38′N | 2012 | N17.5 | 0.363 |  |  |
| 375 | Dima Chen | Typical steppe | 116°42´E | 43°38′N | 2012 | N28 | 0.528 |  |  |
| 376 | XingLiang Xu | alpine meadow | 101°02´E | 37°52´N | 2000 | N0.44 | -0.017 |  |  |
| 377 | XingLiang Xu | alpine meadow | 101°02´E | 37°52´N | 2000 | N0.56 | 0.086 |  |  |
| 378 | Toshihiko Kinugasa | dry steppe | 105°57.08′ E | 47°02′77″N | 2006 | N0.3 | -0.036 |  |  |
| 379 | Toshihiko Kinugasa | dry steppe | 105°57.08′ E | 47°02′77″N | 2006 | N1.5 | 0.069 |  |  |
| 380 | Toshihiko Kinugasa | dry steppe | 105°57.08′ E | 47°02′77″N | 2007 | N0.3 | 0.288 |  |  |
| 381 | Toshihiko Kinugasa | dry steppe | 105°57.08′ E | 47°02′77″N | 2007 | N1.5 | 0.773 |  |  |
| 382 | Toshihiko Kinugasa | dry steppe | 105°57.08′ E | 47°02′77″N | 2008 | N0.3 | 0.053 |  |  |
| 383 | Toshihiko Kinugasa | dry steppe | 105°57.08′ E | 47°02′77″N | 2008 | N7.5 | 0.09 |  |  |
| 384 | Toshihiko Kinugasa | dry steppe | 105°57.08′ E | 47°02′77″N | 2009 | N0.3 | 0.073 |  |  |
| 385 | Toshihiko Kinugasa | dry steppe | 105°57.08′ E | 47°02′77″N | 2009 | N7.5 | 0.221 |  |  |
| 386 | Toshihiko Kinugasa | dry steppe | 105°57.08′ E | 47°02′77″N | 2010 | N0.3 | 0.341 |  |  |
| 387 | Toshihiko Kinugasa | dry steppe | 105°57.08′ E | 47°02′77″N | 2010 | N7.5 | 0.327 |  |  |
| 388 | Xiao Ying Gong | temperate steppe | 115°32´-117°12´E | 43°26′–44°29′N | 2007 | N2.5 | 0.29 |  |  |
| 389 | Xiao Ying Gong | temperate steppe | 115°32´-117°12´E | 43°26′–44°29′N | 2007 | N5 | 0.357 |  |  |
| 390 | Weixing Liu | typical steppe | 116°17´E | 42°02´N | 2005 | N1 | 0 |  |  |
| 391 | Weixing Liu | typical steppe | 116°17´E | 42°02´N | 2005 | N2 | 0.204 |  |  |
| 392 | Weixing Liu | typical steppe | 116°17´E | 42°02´N | 2005 | N4 | 0.316 |  |  |
| 393 | Weixing Liu | typical steppe | 116°17´E | 42°02´N | 2005 | N8 | 0.304 |  |  |
| 394 | Weixing Liu | typical steppe | 116°17´E | 42°02´N | 2005 | N16 | 0.373 |  |  |
| 395 | Weixing Liu | typical steppe | 116°17´E | 42°02´N | 2005 | N32 | 0.373 |  |  |
| 396 | Weixing Liu | typical steppe | 116°17´E | 42°02´N | 2005 | N64 | 0.478 |  |  |
| 397 | Weixing Liu | typical steppe | 116°17´E | 42°02´N | 2006 | N1 | 0.194 |  |  |
| 398 | Weixing Liu | typical steppe | 116°17´E | 42°02´N | 2006 | N2 | 0.24 |  |  |
| 399 | Weixing Liu | typical steppe | 116°17´E | 42°02´N | 2006 | N4 | 0.604 |  |  |
| 400 | Weixing Liu | typical steppe | 116°17´E | 42°02´N | 2006 | N8 | 0.851 |  |  |
| 401 | Weixing Liu | typical steppe | 116°17´E | 42°02´N | 2007 | N1 | -0.085 |  |  |
| 402 | Weixing Liu | typical steppe | 116°17´E | 42°02´N | 2007 | N2 | -0.108 |  |  |
| 403 | Weixing Liu | typical steppe | 116°17´E | 42°02´N | 2007 | N4 | -0.178 |  |  |
| 404 | Weixing Liu | typical steppe | 116°17´E | 42°02´N | 2007 | N8 | 0 |  |  |
| 405 | Weixing Liu | typical steppe | 116°17´E | 42°02´N | 2007 | N16 | -0.042 |  |  |
| 406 | Weixing Liu | typical steppe | 116°17´E | 42°02´N | 2007 | N32 | 0 |  |  |
| 407 | Weixing Liu | typical steppe | 116°17´E | 42°02´N | 2007 | N64 | 0 |  |  |
| 408 | Xiaoxia Yang | Alpine Grassland | 101°12´-101°23´E | 37°29´-37°45´N | 2012 | N10 | 0.335 |  |  |
| 409 | Xiaoxia Yang | Alpine Grassland | 101°12´-101°23´E | 37°29´-37°45´N | 2012 | N10P | 0.115 |  |  |
| 410 | Tao Zhang | Temperate Meadow | 123°45E | 44°45`N | 2006 | N10 | 0.289 |  |  |
| 411 | Tao Zhang | Temperate Meadow | 123°45E | 44°45`N | 2007 | N10 | 0.145 |  |  |
| 412 | Tao Zhang | Temperate Meadow | 123°45E | 44°45`N | 2008 | N10 | 0.12 |  |  |
| 413 | Tao Zhang | Temperate Meadow | 123°45E | 44°45`N | 2009 | N10 | 0.082 |  |  |
| 414 | Liming Yan | Typical steppe | 116°41’E | 42°27′N | 2006 | N28 | 0.516 | 0.873 | 0.258 |
| 415 | Liming Yan | Typical steppe | 116°41’E | 42°27′N | 2006 | N28W | 0.515 | 0.694 | 0.362 |
| 416 | Liming Yan | Typical steppe | 116°41’E | 42°27′N | 2007 | N28 | 0.237 | 0.858 | -0.142 |
| 417 | Liming Yan | Typical steppe | 116°41’E | 42°27′N | 2007 | N28W | 0.244 | 0.769 | -0.304 |
| 418 | Jianyang Xia | Typical steppe | 116°17´E | 42°02´N | 2006 | N10 | 0.266 | 0.182 | 0.296 |
| 419 | Jianyang Xia | Typical steppe | 116°17´E | 42°02´N | 2007 | N10 | -0.082 | 0.526 | -0.163 |
| 420 | Qiuying Tian | Typical steppe | 116°17´E | 42°02´N | 2012 | N2 | 0.155 |  |  |
| 421 | Qiuying Tian | Typical steppe | 116°17´E | 42°02´N | 2012 | N4 | 0.284 |  |  |
| 422 | Qiuying Tian | Typical steppe | 116°17´E | 42°02´N | 2012 | N8 | 0.398 |  |  |
| 423 | Qiuying Tian | Typical steppe | 116°17´E | 42°02´N | 2012 | N16 | 0.458 |  |  |
| 424 | Qiuying Tian | Typical steppe | 116°17´E | 42°02´N | 2012 | N32 | 0.388 |  |  |
| 425 | Jing Jiang | alpine meadow | 91°05´E | 30°51´N | 2010 | N5 | 0.14 |  |  |
| 426 | Jing Jiang | alpine meadow | 91°05´E | 30°51´N | 2010 | N10 | -0.002 |  |  |
| 427 | Jing Jiang | alpine meadow | 91°05´E | 30°51´N | 2011 | N5 | 0.318 |  |  |
| 428 | Jing Jiang | alpine meadow | 91°05´E | 30°51´N | 2011 | N10 | -0.027 |  |  |
| 429 | Jing Jiang | alpine meadow | 91°05´E | 30°51´N | 2012 | N5 | 0.054 |  |  |
| 430 | Jing Jiang | alpine meadow | 91°05´E | 30°51´N | 2012 | N10 | -0.043 |  |  |
| 431 | Liyuan He | Typical steppe | 116°17´E | 42°02′N | 2012 | N10 | 0.406 | 0.17 | 0.587 |
| 432 | Liyuan He | Typical steppe | 116°17´E | 42°02′N | 2013 | N40 | 0.493 | 0.262 | 0.746 |
| 433 | Liyuan He | Typical steppe | 116°17´E | 42°02′N | 2012 | N10 | 0.524 | 0.671 | 0.521 |
| 434 | Liyuan He | Typical steppe | 116°17´E | 42°02′N | 2012 | N40 | 0.648 | 0.847 | 0.74 |
| 435 | Haiyan Ren | Typical steppe | 116°17´E | 42°02´N | 2007 | N10 | 0.043 | 0.842 | 1.072 |
| 436 | Haiyan Ren | Typical steppe | 116°17´E | 42°02´N | 2007 | N10W | 0.212 | 0.464 | 0.656 |
| 437 | Haiyan Ren | Typical steppe | 116°17´E | 42°02´N | 2007 | N10 | 0.256 | 0.4 | 0.05 |
| 438 | Haiyan Ren | Typical steppe | 116°17´E | 42°02´N | 2007 | N10W | 0.343 | -0.743 | -0.577 |
| 439 | Xiangwei Han |  | 110°22´14´´E | 38︒47'25.6"N | 2008 | N2.5 | 0.195 |  |  |
| 440 | Xiangwei Han |  | 110°22´14´´E | 38︒47'25.6"N | 2008 | N5 | 0.219 |  |  |
| 441 | Xiangwei Han |  | 110°22´14´´E | 38︒47'25.6"N | 2008 | N10 | 0.243 |  |  |
| 442 | Xiangwei Han |  | 110°22´14´´E | 38︒47'25.6"N | 2008 | N10P3 | 0.277 |  |  |
| 443 | Wenxia Cao | alpine meadow | 102°31'58.8" | 37°40'1.2" | 2013 | N7.72 | 0.048 | 0.096 | -0.186 |
| 444 | Wenxia Cao | alpine meadow | 102°31'58.8" | 37°40'1.2" | 2013 | N11.57 | 0.022 | 0.061 | -0.164 |
| 445 | Wenxia Cao | alpine meadow | 102°31'58.8" | 37°40'1.2" | 2013 | N19.29 | 0.232 | 0.318 | -0.239 |
| 446 | Wenxia Cao | alpine meadow | 102°31'58.8" | 37°40'1.2" | 2013 | N27 | 0.309 | 0.42 | -0.366 |
| 447 | Wenxia Cao | alpine meadow | 102°31'58.8" | 37°40'1.2" | 2013 | N30.86 | 0.557 | 0.695 | -0.422 |
| 448 | Wenxia Cao | alpine meadow | 102°31'58.8" | 37°40'1.2" | 2013 | N38.58 | 0.402 | 0.516 | -0.307 |
| 449 | Wenxia Cao | alpine meadow | 102°31'58.8" | 37°40'1.2" | 2013 | N46.3 | 0.341 | 0.448 | -0.294 |
| 450 | Wenxia Cao | alpine meadow | 102°31'58.8" | 37°40'1.2" | 2014 | N7.72 | 0.156 | 0.333 | -0.256 |
| 451 | Wenxia Cao | alpine meadow | 102°31'58.8" | 37°40'1.2" | 2014 | N11.57 | 0.145 | 0.317 | -0.253 |
| 452 | Wenxia Cao | alpine meadow | 102°31'58.8" | 37°40'1.2" | 2014 | N19.29 | 0.293 | 0.573 | -0.538 |
| 453 | Wenxia Cao | alpine meadow | 102°31'58.8" | 37°40'1.2" | 2014 | N27 | 0.325 | 0.675 | -0.989 |
| 454 | Wenxia Cao | alpine meadow | 102°31'58.8" | 37°40'1.2" | 2014 | N30.86 | 0.419 | 0.78 | -1.012 |
| 455 | Wenxia Cao | alpine meadow | 102°31'58.8" | 37°40'1.2" | 2014 | N38.58 | 0.457 | 0.861 | -1.59 |
| 456 | Wenxia Cao | alpine meadow | 102°31'58.8" | 37°40'1.2" | 2014 | N46.3 | 0.294 | 0.61 | -0.76 |
| 457 | L. M. HUFF | Forest steppe | 78°87´W | 42°87´N | 2010 | N10 | 0.076 |  |  |
| 458 | L. M. HUFF | Forest steppe | 78°87´W | 42°87´N | 2010 | N10P | 0.172 |  |  |
| 459 | Laura M. Ladwig | desert grassland |  |  | 2004 | N5 | 0.358 | 0.466 | 0.262 |
| 460 | Laura M. Ladwig | desert grassland |  |  | 2005 | N5 | 0.046 | 0 | 0.063 |
| 461 | Laura M. Ladwig | desert grassland |  |  | 2006 | N5 | 0.792 | 0.518 | 1.03 |
| 462 | Laura M. Ladwig | desert grassland |  |  | 2007 | N5 | 0.277 | -0.09 | 0.679 |
| 463 | Laura M. Ladwig | desert grassland |  |  | 2008 | N5 | 0.194 | 0.05 | 0.693 |
| 464 | Laura M. Ladwig | desert grassland |  |  | 2009 | N5 | 0.22 | 0.241 | 0 |
| 465 | Eric G. Lamb |  | 111°33´W | 53°05´N | 2004 | N5.44 | 0.314 |  |  |
| 466 | Eric G. Lamb |  | 111°33´W | 53°05´N | 2004 | N5.44W | 0.222 |  |  |
| 467 | P. GROGAN | savannah | 121°17´W | 39°15´N | 1995 | N20 | -0.173 |  |  |
| 468 | P. GROGAN | savannah | 121°17´W | 39°15´N | 1995 | N20P | 0.024 |  |  |
| 469 | Michal Hejcman | alpine grassland |  |  | 2004 | N5 | 0.2 | 0.331 | -0.036 |
| 470 | Michal Hejcman | alpine grassland |  |  | 2004 | N25 | 0.085 | 0.419 | 0.305 |
| 471 | Michal Hejcman | alpine grassland |  |  | 2004 | N50 | 0.033 | 0.31 | -0.624 |
| 472 | Joke Van den Berge |  | 4°24′E | 51°09´N | 2007 | N1 | 0.192 |  |  |
| 473 | Joke Van den Berge |  | 4°24′E | 51°09´N | 2007 | N3 | -0.05 |  |  |
| 474 | Joke Van den Berge |  | 4°24′E | 51°09´N | 2007 | N7 | 0.032 |  |  |
| 475 | Joke Van den Berge |  | 4°24′E | 51°09´N | 2007 | N15 | 0.068 |  |  |
| 476 | Carly J |  | 7°007´W | 50°199´N | 2009 | N2.5 | -0.263 |  |  |
| 477 | Carly J |  | 7°007´W | 50°199´N | 2009 | N5 | 0 |  |  |
| 478 | Carly J |  | 7°007´W | 50°199´N | 2009 | N10 | 0.182 |  |  |
| 479 | Carly J |  | 7°007´W | 50°199´N | 2009 | N20 | 0.243 |  |  |
| 480 | Andrew H |  |  |  | 2003 | N67 | 0.228 |  |  |
| 481 | Andrew H |  |  |  | 2003 | N67P | 0.114 |  |  |
| 482 | M. Hejcman |  | 13°51´E | 50°12´N | 2007 | N30 | 0.472 |  |  |
| 483 | M. Hejcman |  | 13°51´E | 50°12´N | 2007 | N30P | 0.468 |  |  |
| 484 | M. Hejcman |  | 13°51´E | 50°12´N | 2008 | N30 | 0.656 |  |  |
| 485 | M. Hejcman |  | 13°51´E | 50°12´N | 2008 | N30P | 0.406 |  |  |
| 486 | M. Hejcman |  | 13°51´E | 50°12´N | 2009 | N30 | 0.617 |  |  |
| 487 | M. Hejcman |  | 13°51´E | 50°12´N | 2009 | N30P | 0.386 |  |  |
| 488 | M. Hejcman |  | 13°51´E | 50°12´N | 2010 | N30 | 0.282 |  |  |
| 489 | M. Hejcman |  | 13°51´E | 50°12´N | 2010 | N30P | 0.175 |  |  |
| 490 | Laura Yahdjian |  | 70°16´W | 45°41´S |  | N5 | 0.178 | 0.406 | 0.009 |
| 491 | W.Stanley Harpole |  | 117.755W | 33.624N |  | N10 | 0.386 |  |  |
| 492 | W.Stanley Harpole |  | 117.755W | 33.624N |  | N10W | 0.436 |  |  |
| 493 | Ludwig F | Savanna | 37°E | 4°S | 1998 | N20 | 0.054 |  |  |
| 494 | Ludwig F | Savanna | 37°E | 4°S | 1998 | N20P | 0.11 |  |  |
| 495 | Ludwig F | Savanna | 37°E | 4°S | 1998 | N20 | 0.085 |  |  |
| 496 | Ludwig F | Savanna | 37°E | 4°S | 1998 | N20P | -0.039 |  |  |
| 497 | Ludwig F | Savanna | 37°E | 4°S | 1999 | N20 | 0.053 |  |  |
| 498 | Ludwig F | Savanna | 37°E | 4°S | 1999 | N20P | -8.000 |  |  |
| 499 | Ludwig F | Savanna | 37°E | 4°S | 1999 | N20 | 0.186 |  |  |
| 500 | Ludwig F | Savanna | 37°E | 4°S | 1999 | N20P | 0.485 |  |  |
| 501 | Chris Joyce | hay meadow | 48.85°E | 48.85°N | 1994 | N30 | 0.135 |  |  |
| 502 | Chris Joyce | hay meadow | 48.85°E | 48.85°N | 1994 | N60 | 0.117 |  |  |
| 503 | Chris Joyce | hay meadow | 48.85°E | 48.85°N | 1994 | N30 | 0.175 |  |  |
| 504 | Chris Joyce | hay meadow | 48.85°E | 48.85°N | 1994 | N60 | 0.131 |  |  |
| 505 | Chris Joyce | hay meadow | 48.85°E | 48.85°N | 1995 | N30 | 0.29 |  |  |
| 506 | Chris Joyce | hay meadow | 48.85°E | 48.85°N | 1995 | N60 | 0.634 |  |  |
| 507 | Chris Joyce | hay meadow | 48.85°E | 48.85°N | 1995 | N30 | -0.152 |  |  |
| 508 | Chris Joyce | hay meadow | 48.85°E | 48.85°N | 1995 | N60 | -0.409 |  |  |
| 509 | Ulo niinemets | Wooded meadow | 23.58°E | 58.6°N | 1998 | N2 | -0.014 |  |  |
| 510 | Ulo niinemets | wooded meadow | 23.58°E | 58.6°N | 1998 | N5 | 0.186 |  |  |
| 511 | Ulo niinemets | wooded meadow | 23.58°E | 58.6°N | 1998 | N10 | 0.185 |  |  |
| 512 | Ulo niinemets | wooded meadow | 23.58°E | 58.6°N | 1998 | N20 | 0.393 |  |  |
| 513 | Ulo niinemets | wooded meadow | 23.58°E | 58.6°N | 1998 | N5P | 0.242 |  |  |
| 514 | Ulo niinemets | wooded meadow | 23.58°E | 58.6°N | 1998 | N20P | 0.482 |  |  |
